# Influence of community scorecards on maternal and newborn health service delivery and utilization

**DOI:** 10.1186/s12939-020-01184-6

**Published:** 2020-11-02

**Authors:** Elizabeth Ekirapa Kiracho, Noel Namuhani, Rebecca Racheal Apolot, Christine Aanyu, Aloysuis Mutebi, Moses Tetui, Suzanne N. Kiwanuka, Faith Adong Ayen, Dennis Mwesige, Ahmed Bumbha, Ligia Paina, David H. Peters

**Affiliations:** 1 Department of Health Policy Planning and Management, Makerere University School of Public Health, P.O.Box 7072, Kampala, Uganda; 2 Makerere University School of Public Health, P.O.Box 7072, Kampala, Uganda; 3 Epidemiology and Global Health Unit, Department of Public Health and Clinical Medicine, Umeå University, 901 87 Umeå, Sweden; 4 District Health Service, Kibuku Local Government, P.O Box 150, Mbale, Uganda; 5 Department of International Health, Johns Hopkins Bloomberg School of Public Health, 615 N. Wolfe Street, Baltimore, MD 21205 USA

**Keywords:** Community score cards, Maternal health, Newborn health, Utilization, Accountability, Uganda

## Abstract

**Introduction:**

The community score card (CSC) is a participatory monitoring and evaluation tool that has been employed to strengthen the mutual accountability of health system and community actors. In this paper we describe the influence of the CSC on selected maternal and newborn service delivery and utilization indicators.

**Methods:**

This was a mixed methods study that used both quantitative and qualitative data collection methods. It was implemented in five sub-counties and one town council in Kibuku district in Uganda. Data was collected through 17 key informant interviews and 10 focus group discussions as well as CSC scoring and stakeholder meeting reports. The repeated measures ANOVA test was used to test for statistical significance. Qualitative data was analyzed manually using content analysis. The analysis about the change pathways was guided by the Wild and Harris dimensions of change framework.

**Results:**

There was an overall improvement in the common indicators across sub-counties in the project area between the 1st and 5th round scores. Almost all the red scores had changed to green or yellow by round five except for availability of drugs and mothers attending Antenatal care (ANC) in the first trimester. There were statistically significant differences in mean scores for men escorting their wives for ante natal care (ANC) (F(4,20) = 5.45, *P* = 0.01), availability of midwives (F(4,16) =5.77, *P* < 0.01), availability of delivery beds (F(4,12) =9.00, P < 0.01) and mothers delivering from traditional birth attendants (TBAs), F(4,16) = 3.86, *p* = 0.02). The qualitative findings suggest that strengthening of citizens’ demand, availability of resources through collaborative problem solving, increased awareness about targeted maternal health services and increased top down performance pressure contributed to positive changes as perceived by community members and their leaders.

**Conclusions and recommendations:**

The community score cards created opportunities for community leaders and communities to work together to identify innovative ways of dealing with the health service delivery and utilization challenges that they face.

Local leaders should encourage the availability of safe spaces for dialogue between communities, health workers and leaders where performance and utilization challenges can be identified and solutions proposed and implemented jointly.

## Introduction

The Community Score Card (CSC) is a participatory social accountability tool designed and used to plan, monitor and evaluate services [1]. It brings the suppliers and consumers of a given health service together to identify and analyze challenges to service delivery and utilization so as to find a common and shared way of addressing the issues identified [2, 3]. Ultimately, the community scorecard aims to empower communities and hold people accountable in delivery and utilization of services.

The CSC has been applied in a number of sectors including health, education, water and sanitation, as well as agriculture to enhance social accountability. It has been applied in United States, Asia and African countries such as Malawi, Ghana, Tanzania, Gambia and Uganda) to improve service delivery, boost community ownership of services and improve equity, access and quality of services [2, 4–7].

The evidence about the use of community score cards is mixed. In some African countries, scorecards have contributed to health service delivery improvement [1]. The changes observed were attributed to enhanced community participation and platforms that created dialogue between service users and providers and held stakeholders accountable [8].

Furthermore, they contributed to improved client-provider relationships, attributed for example in Ethiopia, to feedback given by communities on health workers attitude towards work and patients in addition to supply-side changes such as an increase in staffing levels [9, 10]. Though the pilots referenced above yielded some evidence that CSCs can improve service delivery, the findings were at times difficult to replicate and fraught by many barriers to scale and sustainability [5, 11–13]. A recent health-focused accountability and transparency program in Tanzania and Indonesia which did not find any significant differences between the control and treatment arms concluded that the paths linking transparency and accountability programs to health outcomes is complex and that succesful implementation that results in improved health outcomes may require more strategic selection of community participants with increased facilitation support and additional community access to material and relational resources [13].

In Uganda, the CSC has been used in different regions to improve the quality of health and education mainly by nongovernmental organizations. In the health sector, it was implemented in the Karamoja region to improve maternal and child health [7] and in Pallisa and Lyantonde districts to improve social accountability and quality of services in health [6]. Although most of these projects were not rigorously evaluated, they generally led to changes in service delivery with improvement in infrastructure such as construction of staff houses, provision of delivery beds and lighting among others [6]. These actions were implemented by the sub-counties which were tasked to budget for activities such as lighting, while communities often contributed resources such as land used to expand the health facilities [6].

Despite the potential positive impact suggested above, the use of CSCs in improving maternal and newborn health (MNH) services remains limited in low and middle income countries which still bear the heaviest burden of maternal mortality. Their implementation has also been hampered by lack of an indepth understanding of the circumstances under which CSC bring about change. In this paper, we describe the influence of the CSC on the delivery and utilization of MNH services, based on our pilot’s implementation in Kibuku district. Additionally, we use the Wild and Harris’ dimensions of change framework (2012) to explain the routes through which CSCs work, improve service delivery and provide explanations for any changes or for the lack thereof [14]. This kind of evidence will be useful for informing efforts to scale up social accountability initiatives.

## Methods

### Study design and study area

This was an empirical study that employed a mixed methods study design. It was implemented by the Future Health Systems Research Program Consortium [15] through a two-year Department for Internal Aid Development (DFID) funded cost extension. The project was implemented in Kibuku district, Uganda in five sub-counties and one town council. The facility scoring was done in the four public health facilities and one private health facility, where the majority of patients sought health care services.

### Study population

Practicing mainly subsistence farming, the total population of Kibuku district is 202,033 [16]. The study population for this paper comprised of district political leaders, district health team (DHT) members, sub-county technical and political leaders, facility in-charges, health workers, communty members (men and women of reproductive age) and leaders, as well as researchers from Makerere University School of Public Health (MakSPH).

### The CSC implementation design

The implementation was led by stakeholders from Kibuku district, notably sub-county chiefs, local council (LC) chairpersons, health unit management committee (HUMC) chair persons, village health team (VHT) members, community development officers (CDOs), sub-county councilors and volunteers. Technical support was provided by the team from MakSPH.

**Fig. 1:**
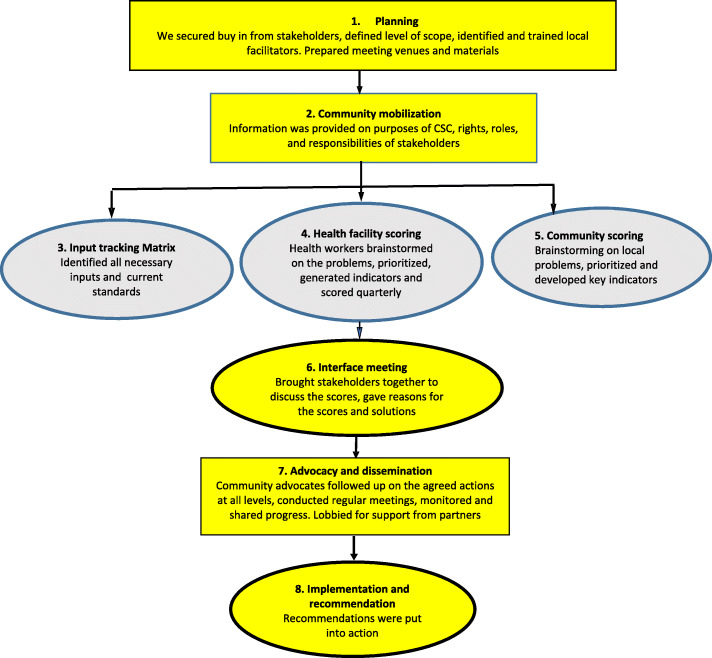
Community score card Implementation design

Five rounds of scoring were implemented on quarterly basis between November 2017 and November 2018. Similar to the CARE CSC design [2], our implementation process included five major steps 1) preparatory ground work (planning, community sensitization and mobilization and input tracking (identification of key inputs for maternal health service delivery) 2) health facility identification and scoring of indicators, 3) community identification, prioritization and scoring of indicators, 4) Interface meeting and 5) dissemination, advocacy and monitoring, as summarized in Fig. 1 .

During the implementation, a few changes were made to the above design in response to feedback from the local stakeholders. During the first round of scoring, one interface meeting was held at the sub-county level, later the interface meetings were held at parish level to allow more people to attend the meetings. After the third round of scoring, we combined the community scoring and interface meetings into one community meeting. This reduced the overall length of the community score card meeting. These meetings were attended by community members, local leaders as well as health care providers. Details about the changes in the design of the CSC can be found in Sebagereka et al. (Additional file 1).

### Data collection methods

#### Quantitative data

Quantitative data was obtained from the data collected during the community score card interface meeting described above. The number of community members at the interface meetings ranged between 20 and 50. It comprised of scores that communities ascribed to supply and demand-side indicators selected and prioritized by communities to assess for changes in performance.

The scores ranged from red (poor performance), to yellow (fair performance), to green (good performance). Six supply and demand side indicators were initially selected by the community at parish level and then later aggregated at the sub-county level and re-grouped based on how frequently they were selected into seven common indicators and six local indicators. The common indicators included mothers attending ANC in 1st trimester, decrease in deliveries attended to by traditional birth attendants (TBA), men escorting partners for ANC, availability of transport to the health facility, availability of midwives, availability of delivery beds and availability of drugs. Five rounds of scoring were done in each subcounty (unit of analysis) and therefore a total of 178 scores were recoded and included in the analysis. The scores were captured by the facilitators (from the district) during the community scoring process and summarized on to manila paper and then later transferred into excel.

#### Qualitative data

With the purpose of triangulating the findings identified through the quantitative scores, the MakSPH team conducted 17 key informant interviews (KIIs) and 10 focus group discussions (FGDs). The interviews were done with purposively selected key informants from the local community, sub-county or district at two time points, 12 months (12 key informants) and 1 year and a half after implementation (five key informants). The study team selected the key informants based on several factors which included their position of leadership at the district or subcounty, their techinal knowledge about maternal and newborn health, their political or religious standing and their involvement in the implementation of the community score card project. The key informants are specified in Table 1.

**Table 1:** Data collection methods

Data Collection Method	Sample size	Period of data collection	Participants
KIIs	17	12 done one year after implementation (June 2018) and 5 after one year and half of implementation (November 2018)	District Development Officers, Sub-county Chiefs, LC Chairpersons, HUMC Chair persons, DHT members, Secretary for Health, RDC and the DISO, Facility in charges and midwives
FGDs	10	One year after the start of implementation (June 2018)	5 FGD’s with 10- 12 men 5 FGD’s with 10-12 women
Review of stakeholder meeting reports	5 meetings with 30- 40 stakeholders	Quarterly after every scoring ( November 2017 to November 2018)	District Development Officers, sub-county Chiefs, LC Chairpersons, HUMC chair persons, DHT members, Secretary for health, RDC and DISO, facility in charges

Ten FGDs (5 FGD’s comprising of women, and 5 FGD’s comprising of men) were randomly selected from the 20 FGDs that were organized for community scoring during the course of each CSC round, after the third round of scoring. Five of the FGDs comprised of 10 women each and another five FGD’s comprised of 10 men each. The FGD paricipants included different categories of community members (women and men of reproductive age, disabled persons, people with different socio economic status, elderly) from the villages in the respective sub-county.

Key informant and focus group guides were used to capture research themes about changes observed, reasons for the changes observed or not observed, facilitators, challenges and barriers. Data was also extracted from CSC project scoring and quarterly stakeholder meeting reports which captured service user and service providers’ perspectives to explain why changes were observed or why changes were not observed.

The data collection methods are summarised in Table 1.

### Data management and analysis

#### Quantitative analysis

The color-coded red, yellow and green designations assigned during the scoring were converted into numerical values 1, 2 and 3, respectively. The average mean scores for each round of scoring was obtained by summing up the scores for the different sub-counties and then dividing by the total number of sub-counties. Sub-counties that did not select particular indicators were excluded during the analysis for such indicators.

We employed a repeated measures one way ANOVA to test for the differences in the mean scores among the five scoring rounds because we are comparing means of scores for more than two groups (rounds) and normality and equality of variances (spherecity) assumptions for repeated measures one way ANOVA were not violated. Bartlett’s test was used to test for equality of variances and Shapiro Wilk test was used to test for normality before running the repeated measures one way ANOVA in STATA 14 [17].

#### Qualitative analysis

The data collected was audio recorded and transcribed verbatim and transcripts coded guided by different themes such as changes in health worker attitudes, improvement in service delivery as well as explanations for the changes. The analysis for the explanations for the changes were guided by the Wild and Harris dimensions of change framework [14] and the theory of change for the project (Additional file 2). Triangulation was done during analysis for each of the indicators. This was done by checking for agreement or disagreement in the findings obtained from the quantitative scores and those from the KIIs and FGDs. Emerging themes were later identified and presented through text, quotes and summary tables.

### Ethical consideration

Ethical approval was obtained from the Makerere University School of Public Health Higher Degrees and Ethics committee and the Uganda National Council for Science and Technology. Written informed consent was obtained from all the key informants, while verbal informed consent was obtained from the FGD participants. All participants were informed that their participation is voluntary and that they were free to stop the interview or FGD at any time. All data collected was anonymous and kept confidential.

## Results

In this paper we present findings on the changes in the common indicators during the five rounds of scoring (Table 2), complemented with perceptions gathered through interviews and FGDs. These gave insights into perceived changes in service utilization and delivery, as well as into why changes in some indicators were more challenging to achieve than others, or why progress in some could not be sustained over the course of the five rounds of implementation. 
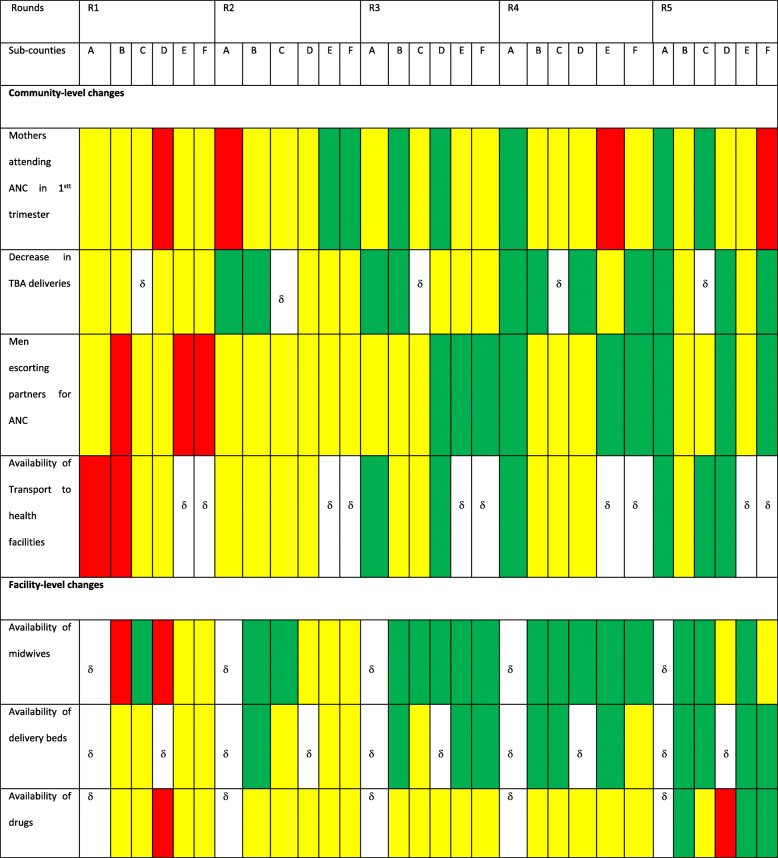

**Table 2:**
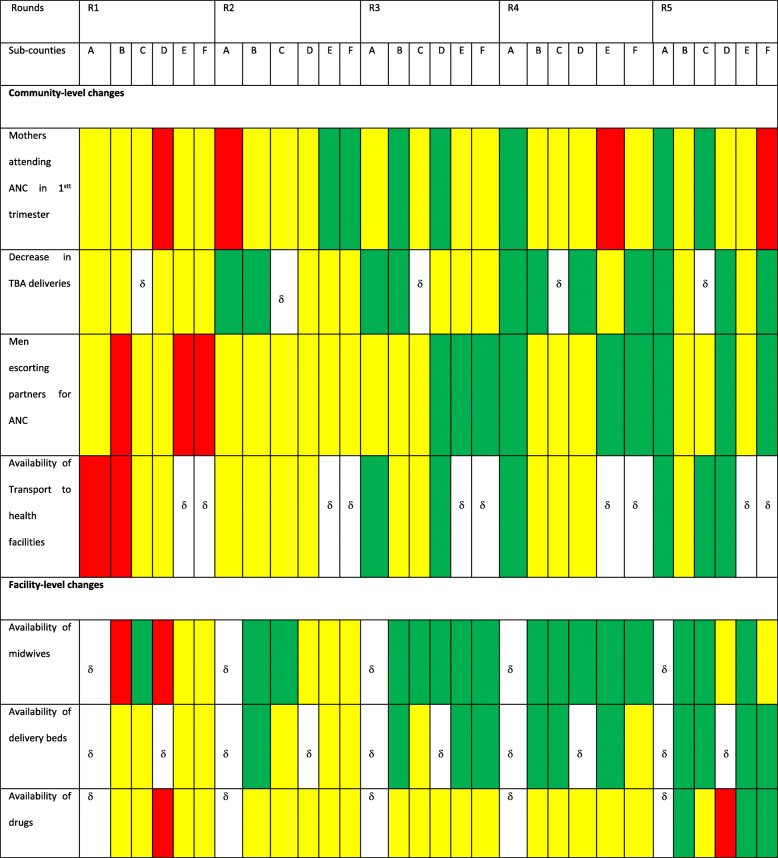
Scores for common indicators across sub-counties

**Key** δ no data was collected because the sub-county did not select this particular indicator

There was an improvement in the common indicators across subcounties in the project area between the first and the 5th round scores. Almost all the red scores had changed to green or yellow by round five except for availability of drugs and mothers attending ANC in the fist trimester where one sub-county still scored red (Table 2). The results from the repeated measures ANOVA can be found in Table 3. Our findings for each indicator are explained alongside the qualitative data that we analyzed.

**Table 3:** Repeated measures one-way ANOVA testing the differences in mean scores during five rounds

	Mean scores per Round (Rd)					Dg. of freedom			
Common indicator	Rd1	Rd2	Rd3	Rd4	Rd5	Rds	Residuals	F	P
**Community-level**									
1^st^ ANC attendance	1.83	2.33	2.33	1.83	2.17	4	20	0.95	0.45
Mothers delivering from TBA	2.00	2.20	2.6	2.8	2.6	4	16	3.86	0.02^*^
Men escorting their wives for ANC	1.50	2.00	2.50	2.5	2.5	4	20	5.45	0.01^*^
Availability of transport to HF	1.50	2.00	2.5	2.25	2.8	4	11	2.76	0.08
**Facility-level**									
Availability of midwives	1.80	2.40	3	3	2.6	4	16	5.77	<0.01^*^
Availability of delivery beds	2.00	2.25	2.75	3	3	4	12	9.00	<0.01^*^
Availability of drugs	1.80	2.00	2	2	2.4	4	16	1.45	0.26

Note: ^*^ Indicates that the value was significant with a *p*<0.05

The changes at community and health facility level are described below;

### Community-level changes

At the community-level, respondents noted perceived improvements in birth preparedness and access to care, though the evidence gathered was mixed in both cases.

### Birth preparedness

Over the five rounds, the scores for men escorting their wives for ANC and for the availability of transport to health facility (HF) increased, though these differences were only significant for the former (F(4,20) = 5.45, *P* = 0.01), and not the latter (F(4, 11) = 2.76, *P* = 0.08). In regards to men escorting their wives, CSC scores indicated that in round 1, three subcounties scored red, this turned to yellow in round 2 and green in round 3 and 4. However scoring in one of the subcounties turned from green to yellow again in round 5. Eight out of 17 of the key informants confirmed positive observations in regards to men being more involved in birth preparedness and support for their wives. These perceptions were held by local leaders, health providers, and community members alike, as illustrated in the quotes below.

*“There has been a change in the mind set of our people especially concerning maternal and newborn health, we can now see that the number of men escorting their wives to the health facility for antenatal and delivery is now increasing.”* (Local political Leader –Sub-county F)

*“I thank the score card […] now when you count out of 10 men you will find that 5 escort women to the health facility, which wasn’t [happening] before.”* (FGD-Women- Sub-county D)

Despite the positive changes reported above, barriers remain. In stakeholder meetings, the low male involvement in some subcounties was attributed to the fact that some men fear that if they go to the facility they will be forced to test for the Human Immuno Defficiency Virus (HIV). This is in response to the nation wide policy that seeks to promote couple counselling and testing for HIV by encouraging all pregnant women to come with their husbands and to get tested for HIV as a couple. Stakeholders also proposed that some men, especially those in gainful employment, do not escort their wives to the antenatal clinics because of the long waiting time. Other men were reported to be influenced by over consumption of alcohol and their peers.

Some of the actions taken by the health workers to encourage male involvement resulted in unintended consequences. For instance some facilities decided to prioritize service provision to women who come with their partners. However, in extreme cases health workers refused to serve women who did not come with their partners. To mitigate this, such women were served if they brought a letter from their local council leader explaining the absence of their husband. However this action also discouraged some men from escorting their wives, they simply decided to obtain a letter. Hence the solution to one problem again created another problem.

### Access to care

Our data also pointed to positive, but mixed improvements in access to MNH care. Over the five rounds, the scores for mothers attending ANC in the 1st trimester improved over time, but not consistently across the five sub-counties. In two subcounties the scores for ANC attendance in the first trimester changed from red in scoring round 1 to green and yellow and then red again in scoring round four. In round five, only two subcounties scored green. Additionally,repeated ANOVA measures showed no statistically significant differences in the mean scores for mothers attending ANC in 1st trimester (F(4, 20), P 0.45).

Nevertheless, more than half of our key informants (10/17) reported a perceived increase in ANC attendance over the course of the CSC implementation as emphasized below;

*“…I will take an example of women who were attending ANC [during] the first trimester, actually the performance wasn’t good by the time we scored first, we were in yellow [50 % of the women were going for ANC early and 50% were going for ANC late], but now we are in green implying that most women, not all but most of them are attending ANC at the facility, this means that the community has picked up the programme and they are [implementing what is recommended]****”*.** (KI Local technical leader -Sub-county A)

*“The implementation of CSC has really helped us because mothers now go for ANC visits from the first month. Mothers have been sensitized to go for ANC visits, to sleep under mosquito nets to avoid malaria in pregnancy and also to go and deliver from the facility so you find that few mothers deliver from the villages.”* (FGD Women-Sub-county B)

However some barriers to ANC attendance were still reported in some of the subcounties. Interface meeting participants reported having a religious sect in a few isolated communities which does not allow its members to seek formal health services, including antenatal care or skilled birth attendance. The local leaders were tasked to talk to the leaders of the sect and to explain to them why it was important for women to attend ANC. In other facilities were ANC attendnace was poor this was attributed to factors such as lack of drugs, long waiting times and poor health worker attitudes.

Regarding the number of women delivering at TBAs, the scoring indicated a change from yellow in four sub-counties in round 1 to green in three sub-counties in the fifth round of scoring with no changes in two sub-counties. Changes in the number of mothers delivering with TBA’s was also statistically signficant (F(4,16) = 3.86, *p* = 0.02). Key informants confirmed that they perceived that fewer women give birth assisted by traditional birth attendants. Multiple local leaders and all focus group commuity participants emphasized this finding.

*“The impact is great, the number or rate of women going to the TBAs has reduced, actually in my sub-county A, I think […] women no longer go to TBAs to deliver, they come to the facility…”* **–**(Local technical leader – Sub-county A)

### Pathways for change at the community level

The analysis for the explanations for the changes were guided by the Wild and Harris dimensions of change framework [14] and the theory of change for the project (Additional file 2). One of the reasons for the changes noted was reported to be ***improved information flow resulting from increased community sensitization***. Respondents suggested that the improved information flow led to increased awareness among stakeholders about their roles, standards, and expected services. For example, more than half (8/17) of the key informants reported that the community became more aware of their rights and responsibilities as a result of the community engagement meetings and sensitizations conducted during the CSC implementation. Respondents believed that greater engagement translated into increased ANC attendances, health facility deliveries and male involvement demonstrated through men escorting their wives to seek care. The key informants at facility level observed that;

*“The scorecard has awakened communities about their health rights and their expectation, let me say their obligations and rights as far as visiting heath facilities is concerned.”* (Facility In charge –Sub-county C)

*“Since they started sensitizing us, we have benefited and we have also gone ahead to sensitize other women and this has [led to more women attending] ANC in the first trimester and then after delivery still they take back their children for PNC (post-natal care).”* (FGD Women Sub-county F).

***Increased citizen demand and collaborative problem solving*** was another reason that was proposed***.*** According to data extracted from the project reports and qualitative interviews, the outcry from citizens about poor health service delivery demonstrated through poor health worker attitudes, lack of basic amenities such as delivery beds, lighting and maternity wards caught the attention of the local leaders. This feedback was acted upon by the leaders who identified different ways of solving the problems identified by partnering with other stakeholders. For example in one facility where construction of the maternity ward had been incomplete, they lobbied the army to complete the construction during their community service week. The community was also called upon to take actions such as joining saving groups to enable them save money for meeting maternal and new born health needs such as transport to the health facility.

The collaborative problem solving described above led to increased availability of resources required for service delivery and improved support for women in pregancy. These changes also influenced health seeking behavouir leading to increased attendance during ANC and delivery.

*“We are now being listened to I can give an example like changes that are now in the health facility is because we voiced out our concerns. [……]it is because our voices were heard that is why health workers have also changed their attitudes.”* (FGD Men – sub-county B)

The community was given a voice to freely speak and hold health workers and local leaders accountable as highlighted by one of the female FGD participants below;

*“Now I have the freedom to speak freely about any problem because even if I went to the hospital and the health worker delays, I just tell her there is nothing, I fear.”* (FGD Women sub-county E).

***The leaders also became more responsive to the local needs.*** The implementation of the CSC was reported by the local leaders to have led to improvements in working relations with district stakeholders, health workers and the community in addition to facilitating more political involvement and support for maternal health services. Seven of the seventeen (7/17) local leaders reported that they perceived improved working relations among local leaders, health workers and the community. Furthermore, there was enhanced political involvement and support for maternal health services demonstrated through increased mobilization and advocacy for increased budget allocations for health. One of the political leaders noted that;

*“Our councilors (local elected political leaders) really had a very negative perspective as far as health issues are concerned, whenever we would tell them lets allocate this kind of money to health, they were really not supportive, but when you people came in, their support to the health budget has improved.”* (KI, Local technical leader sub-county B )

### Changes at the health facility level

#### Availability of midwives and improved health worker attitudes

The scores demonstrated a positive change in the availability of midwives, with only two sub-counties remaining yellow in round 5. This change was also found to be statistically significant (F(4,16) =5.77, *P* < 0.01). This was in agreement with data from key informants and FGDs. Some of the key informants reported a change in the availability of health workers at facilities. A health centre II which used not to have a midwife also received a midwife because the community complained about the absence of a midwife despite the high number of deliveries at the health facility. These issues were mentioned by 5/17 of key informnants and some FGD participants as empasized below;

*“I see now like in our health facility of Goligoli there was only one midwife and he was a male but now we have female midwives and the male ones as well.”* (FGD-Male-sub-county A).

Majority (11/17) of the key informants across all the sub-counties reported that the implementation of the CSC resulted in a positive change in attitudes of midwives towards mothers. Most of the participants in all the FGDs also reported a positive change in attitudes of health workers towards mothers. One of the female participants in the FGD and a district health team member attested to this as echoed below;

*“Previously they [health workers] used to mistreat us but since the CSC implementation things have changed. I was there at 9:00 pm but to tell you the truth, this midwife did not sleep the whole night. She cared about us and even when the mother I was taking care of delivered, […….]she said we should first wait until the baby is immunized.”* (FGD Women-sub-county F)

“*We have seen an improvement actually a dramatic improvement in the attitude of health workers towards clients…*” (District Health team member –sub-county E)

#### Availability of delivery beds

Availability of delivery beds changed from yellow to green in all the four sub-counties where it had been noted to be a problem. This was also statistically significant according to the ANOVA results (F(4,12) =9.00, *P* < 0.01). However, the qualitative results showed that even if the number of beds were reported to have increased, they were not adequate in some of the health facilities.

#### Availability of drugs

Availability of drugs is one of the indicators that did not change immediately. Four sub counties scored it yellow in scoring round one and it remained yellow until scoring round 5 when it changed to green. However in one of the sub-counties it remained red even in the last scoring round. The ANOVA results also reported no significant change. Two key informants said that the problem of limited drugs remained a challenge. One of the FGD participants also attested to this.

*“The problem I have met when you reach the health facility, the health workers welcome you but you leave without getting drugs, they will just tell you; go and buy and what frightens me the most is they tell you we don’t have needles/injection please go and buy them.”* (FGD Men –sub-county C)

Inadequate follow up of implementation of work plans was reported by one of the midwives interviewed as a problem that affected accomplishment of certain targets.

*“Sometimes when these proposals like work plans are made in various meetings, normally there are no follow ups because every time…, there is something new being planned.”* (Midwife-sub-county E)

Some changes were also reported with regard to the functionality of health facilities, where a maternity ward was completed in one of the health facilities and a theatre at the Health Centre IV made more functional. The improved functionality of the health centre IV, was captured in the quote below.

*“…because as I talk now at the health center before this programme came, the health center IV was referring each and every case to Mbale Referral Hospital but as I talk now, most of the surgeries are made here because there was emphasis made on the theatre here to be functional.”* (Political leader sub-county E)

However, the facility level changes noted above were also not without unintended consequences. One of the key informants reported that some staffs were overwhelmed due to increased utilization of services. It created a lot of pressure and increased the workload of the few midwives in health facilities.

### Pathways for change at the facility level

The idea of ***holding stakeholders accountable for their actions or inactions*** was highlighted to be a determinant for change especially in health worker attitudes and practices. During the stakeholder review meetings, the health facility in charges were often asked to explain poor performance. They were therefore more inclined to take actions to adress problems that had been identified. For example, although some facilities had no lighting while others lacked adequate delivery beds, these issues had never been addressed. The heightened attention that was given to these issues in the presence of local leaders linked to different technical and political decision-making platforms, triggered the required actions from the leaders, especially in cases where they had influence.

*“... in fact, before the project came in my sub-county, we were not so familiar with the health facility worker’s problems but as they raised them in the score card, we came to know them and we realized they are genuine problems which needed our immediate attention and we came in.”* (KI, Local political leader sub-county C)

***Top down pressure from leaders for improved performance*** also encouraged the facilities to put in place local initiatives that they felt could improve services. For example in some facilities patients used to wait for a very long time during antenatal care because the facility was congested with too many patients. They therefore decided to implement a set of strategies that would encourage patients to seek ANC through out the week instead of crowding the facility on one particular day. Furthermore the CSC dialogue meetings between the community and the health workers also stimulated them to improve the services that they offered. They were keen to play their role to improve service delivery. This contributed to changes such as improved health worker attitudes.

## Discussion

This study describes both positive and negative changes that were observed at community and facility levels in Kibuku district after 5 rounds of implementation of the CSC over a period of about 18 months. Similar changes in health service delivery have been reported in other CSC projects in countries like Malawi, Gambia and Afghanistan [2–4].The reasons that were proposed as possible explanations for the changes observed, were in line with some of the mechanisms proposed by Wild and Harris as pathways for change using CSCs [14]. They included improved information flow as a result of increased community sensitization, increased citizen demand and collaborative problem solving and finally top down performance pressure. We noted that the availability of safe and open spaces for dialogue between community members, health workers and local leaders is important for promoting increased information flow. Unfortunately, spaces for regular dialogue and interaction between communities, leaders and health workers are limited in many low-income settings (Additional file 1) [17]. In Uganda, existing spaces such as funeral meetings and religious meetings which were successfully used in some of the communities could be used to pass on key health messages to the community. The use of these spaces require minimal additional funding. Furthermore, they do not require special mobilization since they are routine community activities. However, creation of the spaces alone will not result in the desired changes if the concerns identified in these spaces are not communicated to effective oversight and management structures which have the capacity and resources to address the issues raised [13].

Similar to other countries such as Ghana and Afghanistan, in this study, the CSCs provided an opportunity for the community to voice their complaints and demand for better services [1, 3]. It also enhanced collaborative problem solving which led to the harnessing of community resources in solving health service related problems [18, 19]. Indeed, when communities identify problems, and have a problem solving mindset with the belief and willingness to solve their problems, they are able to identify locally appropriate methods and resources for dealing with them [20]*.* In addition they are able to attract the attention of other implementing partners and the support of the sub-county and district councils which control local budgets [21]. This is in keeping with recent accountability work in Tanzania which emphasizes the importance of providing additional material and relational support as well as facilitation if community driven accountability programmes are to lead to changes in health outcomes [13].

It is also critical to note however that CSCs may not only lead to positive actions, but can result in unintended negative actions or tension in the community. This implies that as communities identify and implement solutions, unintended consequences should be anticipated and dealt with as they emerge [22]. It also demonstrates the complex dynamic nature of interacting with communities and emphasizes the need for continuous dialogue and problem solving when utilizing such approaches. Furthermore, it highlights the importance of considering all stakeholders as partners, and identifying more collaborative ways of solving problems rather than finger pointing and punishment especially in the initial phases of action planning during CSC implementation.

Top down pressure from leaders was another reason that was given for the changes observed. The feedback and stakeholder meetings held as part of the CSC process provided spaces in which technical leaders were held accountable by the political leaders for poor performance or took action to solve problems identified. We identified two key lessons from this. Failure to hold technical health managers accountable by the political and sub-county leaders was partly attributed to limited understanding about their role in improving service delivery and a preoccupation of demanding for financial accountability with little emphasis on performance accountability. The orientation meetings held prior to the CSC process and the quarterly stakeholder meetings in which the need to hold technical leaders accountable was emphasized helped to turn this around. However, such efforts are short lived consequently inbuilt routine mechanisms for promoting performance accountability at sub national levels is pivotal for sustaining improved performance accountability [12].

### Methodological considerations and limitations

It is important to note that whereas CSCs allow us to measure community perceptions of change, more accurate measurements of these changes would require more objective forms of measurement. For example, suggested changes in ANC attendance could be ascertained through health facility records or community surveys. Additionally, the repeated measures ANOVA results should be interpreted with caution since the community scoring was done by different community members. However, since they were from the same community and are served by the same facilities, we believe that their views were representative of the community views.

Just like any other community program, measuring change due to CSC implementation is complex. This is because maternal health outcomes happen over a long period of time. Therefore, the time period for this study may not have been adequate to allow us to capture such changes in the 18 months of this project. Quarterly measurement of change could also have been a limitation to this intervention since some of the recommendations for example; building maternity wards needed at least one financial year to implement.

Future research could investigate how to link CSC processes at community level with higher level decision making processes as well as feasibility of implementing such projects at scale and institutionalizing the key processes.

## Conclusions and recommendations

Working with community leaders and communities can enable them to identify local innovative ways of dealing with the health service delivery and utilization challenges that they face. Such innovativeness needs to be harnessed by allowing them to be drivers in the change process and not passive participants. These changes also demonstrate that for positive change to occur the CSC projects need to be implemented in an environment where there are safe and open places for routine dialogue between the community, health facility providers and local leaders. However, there needs to be a mechanism for communicating the stakeholder concerns and recommendations from such dialogues to effective governance structures which can identify and harness locally existing solutions and resources needed to address the problems identified in addition to holding providers concerned accountable for their performance.

To sustain the increased technical and political oversight observed in this study, District and sub-county councils should be given adequate orientation about their role in providing oversight with regard to health service delivery. Furthermore, mechanisms should be put in place to promote performance accountability including regular safe spaces where performance is reviewed and poor performance accounted for by the responsible authorities.

## Supplementary information

**Additional file 1.**Estimating the Cost of Implementing a Facility and Community Score Card to Improve Utilization and Quality of Maternal and Newborn Care Services in a Rural District in Uganda.

**Additional file 2.**Designing for Scale and Taking Scale to Account: Lessons from a community score card project in Uganda.

## Data Availability

The datasets used and/or analyzed during the current study are available from the corresponding author on reasonable request.

## Abbreviations

ANC: Antenatal Care
CDOs: Community Development Officers
CSC: Community Score Card
DHT: District Health Team
DISO: District Internal Security Officer
FGD: Focus Group Discussion
HC: Health Centre
HF: Health Facility
HIV: Human Immunodeficiency virus
HUMC: Health Unit Management Committee
KI: Key Informant
KII: Key Informant Interviews
LC: Local Council
MakSPH: Makerere University School of Public Health
MNH: Maternal and Newborn Health
PNC: Post Natal Care
RDC: Resident District Commissioner
TBA: Traditional Birth Attendant
VHT: Village Health Team
