# Supplementary material for: Influence of community scorecards on maternal and newborn health service delivery and utilization
Source: Int J Equity Health. 2020 Nov 2;19:145. doi: 10.1186/s12939-020-01184-6 (PMC7604954; doi:10.1186/s12939-020-01184-6)
Supplement: Supplementary file 1 — Additional file 1.Estimating the Cost of Implementing a Facility and Community Score Card to Improve Utilization and Quality of Maternal and Newborn Care Services in a Rural District in Uganda. [file 12939_2020_1184_MOESM1_ESM.pdf]

International Journal for Equity in Health

Estimating the Cost of Implementing a Facility and Community Score Card to Improve Utilization and Quality of Maternal and Newborn Care Services in a Rural District in Uganda

--Manuscript Draft--

**Manuscript Number:**	
**Full Title:**	Estimating the Cost of Implementing a Facility and Community Score Card to Improve Utilization and Quality of Maternal and Newborn Care Services in a Rural District in Uganda
**Article Type:**	Research
**Funding Information:**	DFID through the Future Health Systems (FHS) Consortium program (HRPC09 Delivering Effective Health Services) — Dr. Elizabeth Ekirapa Kiracho

Abstract:

Introduction

This paper aimed at estimating the resources required to implement a community scorecard by a typical rural district health team in Uganda, as a mechanism for fostering accountability, utilization and quality of maternal and child healthcare service.

Methods

This costing analysis was done from the payer's perspective using the ingredients approach over five quarterly rounds of scoring between 2017 and 2018. Expenditure data was obtained from project records, entered and analyzed in Microsoft excel. Two scale-up scenarios, scenario one (considered cost inputs by the MakSPH research teams) and scenario two (considering cost inputs based on contextual knowledge from district implementing teams), were simulated to better understand the cost implications of integrating the Community Scorecard (CSC) into a district health system.

Results

The total and average cost of implementing CSC for five quarterly rounds over a period of 18 months were USD 59,962 and USD 11,992 per round of scoring, respectively. Out of a total of six sub-counties (including one Town Council) considered in this analysis, the average cost per sub-county and scoring round was USD 1,998. Scaling-up of the intervention across the entire district (included 22 of sub-counties) under the first scenario would cost a total of USD 19,003 per scoring round. Under the second scaleup scenario, the cost would be lower at USD 7,116. The total annual cost of scaling CSC in the entire district would be USD 76,012 under scenario one compared to USD 28,465 under scenario two. The main cost drivers were transportation costs, coordination and supervision costs, and technical support to supplement local implementers.

Conclusion

Our analysis suggests that it is financially feasible to implement and scale-up the CSC initiative, as an accountability tool for enhancing service delivery. However, the CSC design and approach needs to be embedded within local systems and implemented in collaboration with existing stakeholders so as to optimise costs. A comprehensive economic analysis of the costs associated with transportation, involvement of the district teams in coordination, supervision as well as provision of technical support is necessary to determine the cost-effectiveness of the CSC approach.

Corresponding Author: Anthony Ssebagereka
Makerere University School of Public Health
Kampala, Uganda, UGANDA

Corresponding Author E-Mail: assebagereka@gmail.com

Powered by Editorial Manager® and ProduXion Manager® from Aries Systems Corporation

**Corresponding Author Secondary Information:**	
**Corresponding Author's Institution:**	Makerere University School of Public Health
**Corresponding Author's Secondary Institution:**	
**First Author:**	Anthony Ssebagereka
**First Author Secondary Information:**	
**Order of Authors:**	Anthony Ssebagereka
	Rebecca Racheal Apolot
	Evelyne Baelvina Nyachwo
	Elizabeth Ekirapa Kiracho
**Order of Authors Secondary Information:**	
**Additional Information:**	
**Question**	**Response**
**Is this study a clinical trial?**<hr><i>A clinical trial is defined by the World Health Organisation as 'any research study that prospectively assigns human participants or groups of humans to one or more health-related interventions to evaluate the effects on health outcomes'.</i>	No
Have you submitted similar articles (including as part of a series or from the same project) to this or any other journals?	Yes
Please provide details of the other submissions below: as follow-up to "Have you submitted similar articles (including as part of a series or from the same project) to this or any other journals? "	Designing for Scale and Taking Scale to Account: Lessons from a community score card project in Uganda Which contextual factors facilitate successful implementation of Community Score Cards in Uganda?

Powered by Editorial Manager® and ProduXion Manager® from Aries Systems Corporation

Manuscript document
Click here to access/download;Manuscript;CSC costing paper - vrsn 1.docx
Click here to view linked References

Estimating the Cost of Implementing a Facility and Community Score Card to Improve Utilization and Quality of Maternal and Newborn Care Services in a Rural District in Uganda

Anthony Ssebagereka[1*], Rebecca Racheal Apolot[1], Evelyne Baelvina Nyachwo[1], Elizabeth Ekirapa-Kiracho[1]

Author affiliations

[1] Department of Health Policy, Planning, and Management, Makerere University School of Public Health, New Mulago Hospital Complex, Kampala, Uganda

[*]Corresponding author

Author affiliations

Anthony Ssebagereka, Department of Health Policy Planning and Management, Makerere University School of Public Health, P.O. Box 7072, Kampala, Uganda. assebagereka@gmail.com (corresponding author)

Evelyne Baelvina Nyachwo, Department of Health Policy Planning and Management, Makerere University School of Public Health, P.O. Box 7072, Kampala, Uganda. enyachwo@gmail.com

Rebecca Racheal Apolot, Department of Health Policy Planning and Management, Makerere University School of Public Health, P.O. Box 7072, Kampala, Uganda. apobecca@gmail.com /apobecca@musph.ac.ug

Elizabeth Ekirapa, Department of Health Policy Planning and Management, Makerere University School of Public Health, P.O. Box 7072, Kampala, Uganda. ekky@musph.ac.ug / ekky01@gmail.com

Abstract

Introduction: This paper aimed at estimating the resources required to implement a community scorecard by a typical rural district health team in Uganda, as a mechanism for fostering accountability, utilization and quality of maternal and child healthcare service.

Methods: This costing analysis was done from the payer's perspective using the ingredients approach over five quarterly rounds of scoring between 2017 and 2018. Expenditure data was obtained from project records, entered and analyzed in Microsoft excel. Two scale-up scenarios, scenario one (considered cost inputs by the MakSPH research teams) and scenario two (considering cost inputs based on contextual knowledge from district implementing teams), were simulated to better understand the cost implications of integrating the Community Scorecard (CSC) into a district health system.

Results: The total and average cost of implementing CSC for five quarterly rounds over a period of 18 months were USD 59,962 and USD 11,992 per round of scoring, respectively. Out of a total of six sub-counties (including one Town Council) considered in this analysis, the average cost per sub-county and scoring round was USD 1,998. Scaling-up of the intervention across the entire district (included 22 of sub-counties) under the first scenario would cost a total of USD 19,003 per scoring round. Under the second scaleup scenario, the cost would be lower at USD 7,116. The total annual cost of scaling CSC in the entire district would be USD 76,012 under scenario one compared to USD 28,465 under scenario two. The main cost drivers were transportation costs, coordination and supervision costs, and technical support to supplement local implementers.

Conclusion: Our analysis suggests that it is financially feasible to implement and scale-up the CSC initiative, as an accountability tool for enhancing service delivery. However, the CSC design and approach needs to be embedded within local systems and implemented in collaboration with existing stakeholders so as to optimise costs. A comprehensive economic analysis of the costs associated with transportation, involvement of the district teams in coordination, supervision as well as provision of technical support is necessary to determine the cost-effectiveness of the CSC approach.

Keywords: Community Scorecard, cost analysis, accountability, health services, maternal, child health

Introduction

Social accountability interventions have been implemented widely, especially in resource-limited settings, to increase accountability and responsiveness to consumers of services by offering a platform for dialogue between consumers and service providers(1).Their ultimate goal is usually to improve accountability and service delivery. Community Scorecards (CSCs) are one of the social accountability mechanisms that have

been employed to improve accountability and responsiveness of service providers. They have been used as a mechanism to promote equity, access, and utilization of health services especially maternal and child health services (2, 3).

Community scorecards improve transparency and community participation in decision making about service delivery by health facilities within their respective communities, which ultimately leads to improved quality of care from a clients' perspective (4-8). The latter is attained through improved patient-provider relationships, improved performance of service providers (including better behaviors by service providers), and local authorities' improved responsiveness in terms of time and resources allocation (1, 9). Furthermore, better information sharing and communication during the CSC implementation catalyzes improvements regarding health workers' responsiveness to clients' service needs(10).

Current evidence and experiences from a number of interventions and pilot studies suggest that CSCs can be a useful tool for improving accountability and governance for health service delivery, quality, equity in access and utilization of health services (11-13). However, the effectiveness of CSC initiatives is dependent on a number of factors; including the commitment and quality of local leadership to support the process, capacity of the implementing organization or institution, as well as characteristics of the local communities where the CSC process is implemented (14, 15).

Uganda is one of the countries where CSCs have been piloted and CSC pilots have contributed to an increase in service utilization indicators such as health facility delivery, family planning and immunization (1, 9, 16, 17). However, these CSC pilots have been implemented in a few districts often by civil society organizations and have not culminated into a national scale-up. One major barrier to full implementation and scale-up of CSCs in Uganda has been limited financial resources to achieve sustainability and efficiency in CSC implementation(18). Documented evidence on the cost of implementing a CSC initiative and the costs of social accountability more broadly is lacking, yet understanding the cost of implementing the CSC process is important in informing discussions and considerations for scale-up, sustainability, and institutionalization of such efforts.

Issues regarding accountability and social accountability are currently a topic of discussion in the policy arena, especially because of the renewed interest in attaining Universal Health Coverage (UHC) and the Sustainable Development Goals (SDGs). Therefore, a costing analysis is particularly timely in Uganda, where institutionalization of community scorecards has not yet been realized despite several pilots. In Financial Year2014/15, the Uganda Ministry of Health developed a national and facility-level score card for Reproductive, Maternal, Newborn, and Child Health (RMNCH) to promote transparency and

accountability in service delivery(19). This CSC provides a model that could be modified and used as a complement to the RMNCH scorecard, in order to add a community engagement component.

In this paper, we use the Future Health Systems' Community Scorecard (FHS-CSC) case study to estimate the resource requirements, including the costs of implementing a CSC as a mechanism to foster social accountability in Maternal and Newborn health service delivery in a typical district health system in Uganda. While the costs themselves might be difficult to generalize in settings outside of Uganda, this paper also describes a costing process that can be adopted to estimate CSC implementation costs for similar contexts and can be adapted for costing of additional social accountability mechanisms.

Methods

Study Area

This costing analysis was part of the FHS-CSC study. The FHS CSC study was conducted in five sub-counties and one Town Council of Kibuku, a rural district in Eastern Uganda. Kibuku district is a relatively new district established in 2010, and is still struggling to establish a robust system to deliver on its mandate. A sub-county is an administrative unit that is served by at least one Health Center III, usually with a catchment area of 30,000 people. Within each sub-county, are lower administrative units called parishes, which usually have a population of about 5,000 residents. Kibuku district has 22 sub counties, 87 parishes, and 402 villages. A Town Council is a peri-urban area usually a major town in a rural district, resident to about 10,000 people. It's worthwhile noting that the median number of sub-counties in each district in Uganda is 11 and this number ranges between 5 to 28 sub counties(20). Thus, considering these aspects, Kibuku district generally represents a typical rural district setting in Uganda. Based on the district's number of administrative units, several CSC scale-up scenarios were later developed, as shown in Table 6.

In Uganda, under the decentralization policy, health service delivery is a mandate of the district local government(21). The district health system is superintended over by the district health office(22) that co-ordinates resource distribution, staff deployment and overall supervision of the health facilities including the district hospital, Health Center IV, IIIs and IIs (21). Kibuku district has a total of 17 public sector health centers, but does not have a government hospital.

Structure of the FHS-Community Scorecard study in Uganda

The CSC entailed a series of facilitated meetings with and between Maternal and Newborn health service providers, users, district local government officials, political leaders, and other stakeholders. The meetings served as a platform for different stakeholders to share feedback regarding service delivery, identify service

delivery and utilization challenges, and jointly work on generating solutions. Overall, the CSC involved a number of key processes as elaborated in Table 1. Further details on the FHS-CSC implementation in Uganda can be found in *Ekirapa-Kiracho et al* (17, 23).

Table 1 here

During the implementation of the community score card, a number of adjustments in the mode of implementation were made, based on a learning-by-doing approach. These adjustments were mainly aimed at optimizing gains in efficiency and effectiveness. The implementation adjustments across the five *scoring rounds* are summarized in Table S1 (supplementary files).

Costs: sources, measurement and perspective

The cost analysis reported in this paper only refers to resources that were used in the implementation of the FHS- CSC intervention for the period June 2017 to December 2018. All costs were reported in United States Dollars (USD) during the year 2018, after making appropriate adjustments. Costs included in this analysis were estimated based on project accounting, financial and administrative records. The analysis adopted a provider's perspective (*payer perspective*); that is, only resources expended on implementation of the FHS-CSC were considered. This perspective was more appropriate for answering the main objective of this analysis – which was mainly to estimate the resource requirements for implementing a CSC within a district health system. A providers' perspective would also be useful in providing insights into resource or budget implications should Ministry of Health or government propose to integrate the program into the district health system or National Health Service delivery system (when scaling up). The value of time spent by the district staff and Makerere University School of Public health (MakSPH) research team was also considered when they contributed to implementation of the CSC.

Costs were classified into a) startup costs, and b) operational costs. The startup costs reflected the preparatory ground work that was carried out before round one of the CSC. These included costs for activities such as trainings, community sensitization, as well as stakeholder mobilization and buy in (Table 1). The operational costs, on the other hand, were incurred while running the CSC activities between round one and round five. The activities included community and facility scoring, interface meetings, district scoring, feedback meetings, stakeholder coordination, supervision, refresher trainings, stakeholder dissemination meetings, follow-up meetings and provision of technical support to the district team.

Program costs were identified and costing done based on the ingredients approach; where all program inputs were identified, quantified or measured, assigned monetary values and allocated (24-26).Costs were classified by major activities as per the CSC implementation design. For each CSC activity, all costs

incurred were identified and specified. The CSC intervention costs were broadly grouped into 10 categories shown in Table 2.

Table 2 Here

Estimating total costs

Costs incurred during the CSC program implementation consisted of both fixed (semi-variable) costs and variable costs. Costs of coordination were semi-variable, in a sense that they had already been determined during the program budgeting process. However, other costs were variable in a sense that they were dependent on contextual aspects and field dynamics; for example, frequency of an activity – such as mobilization and sensitization, trainings and capacity building costs, among others. While the CSC outcome indicators included improvements in service utilization, we were unable to estimate the unit costs based on this indicator because the intervention did not have a control area to determine attribution of effect to the program. After obtaining the costs of the individual program inputs, these costs were aggregated to determine the total program cost over the intervention period (27). We calculated the total and mean cost of implementing the CSC per round. The CSC round was selected as the unit of analysis due to its programmatic significance at implementation level; for example, in case a district wants to implement a given number of CSC rounds, findings from this study can readily inform planning and budgeting efforts for a successful activity. No discounting was done, because the implementation costs were incurred virtually within a one-year time horizon. All costs were captured in Uganda Shillings (UGX) and reported in USD. A conversion rate of UGX 3600 per USD was used during the analysis.

In order to examine the overall cost drivers, we calculated costs per CSC component, and conducted simulations for two scale-up scenarios, discussed in the following section below. Scenario one was proposed by, and from the perspective of the MakSPH research team, while the other scenario was from the District implementation teams considering no support from the MakSPH team. Subsequently, the two scenarios were rigorously discussed upon with regard to the possibilities of scaling the intervention to the entire district, and consensus was reached on having simulations of the two scenarios.

Simulating CSC scale-up scenarios.

We simulated two scale up scenarios broadly based on both the geographical and time elements. This was to demonstrate possible implications if the pilot were to be scaled-up to other 22 sub-counties, and also implemented in the entire Kibuku district over a period of one year beyond the period of the FHS project. During the simulation, we did not adjust for inflation or discount any costs since the implementation costs were incurred virtually within a one-year time horizon. Furthermore, these scenarios aim to depict

implications on overall cost when key cost drivers are adjusted to enhance efficiency and effectiveness. The scenarios factor in feasibility and cost considerations as main decision-making criteria that Ministry of Health officials and District Health Teams might use for decision-making regarding implementation of the CSC. The components of what was included in the scale up scenarios as well as explanations for inclusion or exclusion are shown in Table 3.

Table 3 here

The CSC scale-up costs for training, mobilization, facilitation and coordination allowances, and for each scenario were estimated, from which we computed the average start up and operational costs; first, for a single scoring round, and then later, calculated the annual costs of CSC implementation. Annual costs were estimated by projecting the costs for four scoring rounds (if scoring was to be done on a quarterly basis). Average cost estimates for implementing a single scoring round as well as the projected annual costs were each categorised in two levels; cost estimates of CSC implementation in one sub-country and then scaleup costs to the entire district.

Scenario one cost inputs were determined by the MakSPH research team (based on the expenditures and experiences of implementation of CSC rounds 3, 4 and 5). In scenario one, the participation of the MakSPH team was reduced to only the three days of the initial training and the costs for other meetings were reduced to reflect government per diem rates. For example, while the project often paid a transport refund of UGX 30,000 (USD 8.3), the government rate was UGX 17,000 (USD 4.7).

The cost inputs into scenario two were decided upon by the sub-county coordinators and District Health Team (based on their experiences of implementing the CSC process during the pilot phase). Hence, this reflects their contextual opinion about the costs they felt would be required to scale up/implement, and fund the community score card under the district health system. In scenario two, we eliminated support from the MakSPH team, except for the initial training. It also substantially reduces the costs incurred for conducting of meetings by either dropping or lowering the per diems paid, shortening the meetings or piggy backing on other meetings.

In our analysis, we took into account the following assumptions:

1) Implementation of the CSC processes was consistent with the protocol guidance given to the implementing teams to achieve evenly distributed optimum outcomes across the subcounties. Furthermore, it was assumed that Kibuku district represented structures, context and operations of a typical rural resource-constrained district.
2) A constant number of people would attend the scoring, preparatory and feedback meetings held at the district and subcounty offices in all the scoring rounds.

3) The payments made to the MakSPH team represented the typical costs that would be incurred by a technical implementing partner for the CSC process.
4) All expected district and subcounty local government meetings would happen as scheduled hence the health facility scoring would be conducted during routine facility review meetings and the district implementation teams (comprised of the DHT, the district and sub-county coordinators) made rational input based on the district local government implementation context regarding implementation of the CSC.
5) The district comprised of the total number of sub counties (22 sub counties for Kibuku) and the average cost of implementation was similar across all subcounties.

This costing analysis complies with the consolidated health economic evaluation reporting standards (CHEERS) checklist, see Table S3 (supplementary files).

Results

Summary costs for the CSC implementation processes

The overall total cost of implementing the CSC intervention in the five sub counties and one town council of Kibuku district was USD 59,962 with an average cost per scoring round of USD 11,992. The average cost of conducting the CSC per subcounty was USD 9,710, while the average cost per round per sub-county was USD 1,998. It is important to note that the implementation modalities were the same across all sub counties hence the costs of implementation were generally comparable across the subcounties. Expenditures for the different CSC implementation activities; preparatory ground work (planning, community sensitization and mobilization and input matrix tracking), health facility scoring, community scoring, district scoring, interface meeting, dissemination and monitoring and evaluation are presented in Table 4.

Table 4 here

The highest implementation costs were incurred during monitoring and evaluation, preparatory ground work and interface meetings. The monitoring and evaluation phase included follow-up meetings at district and sub-county levels as well as feedback meetings with the CSC implementation team. However, substantial costs were also attributed to allowances for per diem and transportation as well as refreshments as highlighted in Table S2 of the additional files. The costs for the community scoring decreased in the 4th and 5th round because the community scoring and interface meetings were combined. However, additional costs were attributed to the interface meeting during both rounds, as compared to the community scoring meetings, and thus, the interface meetings had higher costs compared to the community scoring. On the other hand, the costs of implementing district scoring and dissemination meeting in round four increased

because there was an increase in the number of participants at these meetings. Lastly the monitoring and evaluation costs dropped in round five because we did not conduct follow up meetings, in order to optimize the limited resources available. Details of the CSC implementation costs are presented in Table S2 of the additional files.

Implementation costs of the CSC

Preparatory ground work was carried out before round one of the CSC and included costs for trainings of the DHT and supervisors/coordinators on CSC, community sensitization, and stakeholder mobilization, coordination, transport, facilitation, stationery and Refreshments. Thus, the total CSC startup costs were USD 11,423. Trainings accounted for the highest proportion of startup costs, accounting for up to 38% (USD 4,300) of the total startup costs, while stationery costs contributed the lowest proportion. Implementation costs are summarised in Table 5.

Table 5 here

Overall, the highest costs were incurred on transportation of technical teams (One third of the total program costs were attributed to transportation for the technical teams), participants to and from the meeting venues, facilitators and supervisors of meetings. Round one had the highest costs because it was the first scoring round and therefore the support from MakSPH was more intense, with more members of the team and hence higher transport costs.

Supervision incurred the second highest costs and this could be attributed to the higher per diem costs for the MakSPH team. Coordination costs were also high, with a lot of activities happening at the community, Sub-county, district in addition to the technical coordination support from MakSPH. The CSC intervention involved a lot of activities and meetings, as detailed in Table 2, and therefore high-level coordination was key to its success. On the other hand, expenditures on stationery were the lowest, with most of the costs being incurred during rounds one and two. The costs of stationery gradually declined in rounds 3, 4 and 5 mainly because the community scoring and interface meeting was combined and low-cost materials used to lower implementation costs.

Scale up scenarios: Sub-county and district level

Two CSC scale-up scenarios were simulated: the first one took into account cost inputs based on experiences of the MakSPH team in providing technical support to the district teams, while the second one took into consideration cost inputs from the district CSC implementation teams, based on local contextual knowledge. For each scenario (Table 6), we present the startup and operational costs for a single scoring

round per subcounty and for the entire district (22 sub counties in the entire district). We also present the annual costs of CSC implementation (four scoring rounds per year) per subcounty and per district.

Table 6 here

Scenario one

The average cost of implementation of a single scoring round per sub-county was USD 1,716 in Scenario one (with average startup costs of USD 1,542 and operational costs of USD 174.4). More strikingly, the total cost of a single scoring round for the entire district was USD 19,003. The annual cost of CSC implementation was USD 76,012 in Kibuku district over the four rounds.

Scenario two

On the other hand, the average cost of implementation of a single scoring round per sub-county was even lower in scenario two at USD 1,002 (with startup costs USD 954 and operational costs of USD 48) compared to the earlier scenario. In addition, the total cost of a single scoring round for the entire district was much lower at USD 7,116 compared to scenario one, while the annual cost of CSC implementation in Kibuku district over the four rounds was USD 28,465.

Discussion

We estimated the cost of implementing a facility-based and community-based scorecard that was aimed at optimizing social accountability in maternal and newborn health service delivery for a typical district in a resource-limited setting. To the best of our knowledge, this is one of the first CSC costing papers in low-income settings like Uganda. The overall total cost of implementing the CSC intervention in five sub counties and one town council in Kibuku district was USD 59,962. Furthermore, the average cost of CSC implementation per scoring round per sub-county was USD 1,998.

The highest CSC implementation costs were incurred during monitoring and evaluation activities, interface and dissemination meetings, and during preparatory ground work. These activities had a fairly high number of stakeholders who received per diem as well as allowances for transport, meals and refreshments. Experience from the CSC implementation shows that expenditures on items such as transport and refreshments could not be avoided in this typical district setting since it was observed that stakeholders are accustomed to receiving allowances for attending meetings. Thus, it was difficult to expect them to participate in the project activities without receiving any allowances (23). However, we noted that there is a window of opportunity for reducing the number of stakeholders and amount of allowances they were paid, which would contribute to a reduction in the overall cost of CSC implementation as observed in the simulations for scenario two.

Although we had expected to lower expenditure on dissemination meetings by piggy backing them to other existing meetings within the district management structures, for example, the sub-county council meetings, the set back to this initiative was that these meetings sometimes did not take place, for instance, when the appropriate line funding was either delayed or not received from the central government. In addition, the agenda paper for the meetings were full in some cases, and would not allow sufficient time to have detailed discussion of issues and challenges identified during the scoring meetings (12, 23). The team, therefore, tended to use such opportunities for joint meetings to provide feedback and to garner support from stakeholders for specific activities.

On the other hand, there were much lower costs incurred in conducting the facility and district meetings. This could be attributed to the small number of participants in these meetings; on average there were 20 members during the district scoring and about 5-10 individuals for the facility meetings. Additionally, only one district meeting was held per quarter and five health facility meetings were convened in each quarter. These meetings therefore culminated into lower costs for the components of transportation and facilitation per diem. It’s important to note that per diems paid to the district participants were higher compared to those of other stakeholders.

When we reviewed the operational costs, we noted that transportation, supervision and coordination attracted the highest costs overall. This was mainly attributed to the higher costs of transportation and per diem for the capital city-based MakSPH technical support team in comparison those given to the district technical support teams. In addition, the MakSPH support team remained actively engaged throughout the five rounds of scoring and this contributed to the overall high costs for the CSC pilot. The CSC intervention involved a lot of activities and therefore high-level coordination was a key component to its success. Although the MakSPH team intended that the District Health Team (DHT) would take over this role, there was low level of involvement of the DHT during the initial CSC rounds, mainly due to their busy work schedules, with majority of the DHT members joining the scoring activities during the third round. In fact, during the first and second scoring rounds, only two DHT members supported the scoring activities. But later on, eight more DHT members joined the team and more coordinators were added to improve the coordination process, resulting in an increment in the supervision and coordination costs. Therefore, during scaleup, careful consideration has to be given to the willingness or extent to which the DHT is involved in the CSC activities, amidst their busy schedules and other competing interests, since these will have a big bearing on the success of the CSC if it is to be implemented entirely within the district health system. Thus, extra technical support has to be given to the DHT in case there is limited level of involvement/support towards the CSC activities and this will have cost implications, especially during the earlier stages. However districts that have civil society organizations that are already involved in similar work may be able to leverage their support with minimal additional costs.

Generally, the main CSC activities, such as the community scoring, required relatively fewer costs because the project team deliberately lowered most of the respective costs, while other cost items like refreshments and transport refund for community participants during scoring rounds three, four and five were eliminated. This decision was arrived at after having realized that providing such facilitation raised the monetary expectations (in a way, setting a precedent) that are difficult to sustain. Furthermore, it undermines the spirit of voluntarism and participation that are major foundations for primary health care. However, it is important to note that such decisions are not always welcomed by community members, mainly because they often expect monetary compensation at the end of meetings (21). In addition, the costs for the community scoring also decreased in the fourth and fifth scoring rounds since scoring was combined with the interface meeting and it took a smaller proportion of the meeting time compared to the other activities and hence lower costs.

Alternatively, interface meetings had higher costs compared to the community scoring and the district scoring. This could be attributed to the higher number of interface meetings (25 meetings) and related costs (refreshments, transport allowances and per diem for facilitation and supervision) per quarter between rounds two and five. Whereas, broad stakeholder engagement is desired to maximize outcomes from the CSC processes, inclusion of a wide range of stakeholders can be time-consuming as well as cost prohibitive(28).Therefore, careful consideration and inclusion of individuals, especially those that add substantial input to the core process is required to optimize resources and outcomes.

When compared to the quarterly unconditional grants from the central government of USD 833 received by each sub-county from which they are required to budget for health, the CSC operational costs appear affordable since under *scenario two,* it amounts to only 5% of the sub-county budget. This argument is also supported by findings from a similar study done in Satara district in India that introduced social accountability mechanisms and found that these CSC activities were quite affordable, accounting for even less than 1% of the district total budget, and yet resulted in substantial achievements including behavior and institutional changes as well as concrete outcomes(29). Similar actions could be taken in Uganda especially if the CSC activities are implemented as part of an integrated package of services. They could also take advantage of the recently launched constituency assembly meetings (Baraza's) which are community advocacy forums for enhancing political and performance accountability(30, 31).

The simulations showed that when CSC activities are implemented and funded largely by local stakeholders, the costs are much lower compared to circumstances where they are implemented and funded by external partners (17, 23). High costs of implementation are one of the factors that constrain the implementation and scale-up of hitherto effective interventions across many settings, and more so for resource constrained settings. However, it is important to note that the overall cost of CSC implementation

is highly dependent on the specific CSC design, context, location, scope, scale, and these factors indeed influence the possibility of scale-up(32).

Strategies need to be adopted to minimize costs, including identifying and minimizing use of inputs that attract high costs. In doing this, however, caution ought to be taken to ensure that essential and key programme inputs are not eliminated leading to ineffective implementation. Some strategies to minimize costs may include limiting allowances in the initial phases (startup), encouraging community effort and participation, and building local or district capacity so as to limit technical support to only critical programme areas where local capacity may still be lacking or reasonably insufficient. It is however important to note, as demonstrated in scenario two, that if local stakeholders receive adequate support in the earlier phases of the project, they may be eventually capable of implementing the community score cards on their own at very minimal cost. High initial or start-up costs may therefore be reduced as the project design is further modified and adapted to the local context.

Some of the limitations of this work include the fact that we have simulated the costs for the scale-up scenario, having not had the opportunity to allow us to capture actual costs, and hence the costs proposed should be interpreted with caution. In addition, the scale-up scenarios are based on costs from one rural district. This could affect the generalizability of our findings to other districts in Uganda especially those that have stronger structures/systems and those located in urban settings with relatively more funding from the central government or donor funded projects. However, Kibuku district is typical of other new rural districts which are still building and setting up local structures. It therefore arguably represents a resource limited context which most districts in Uganda face and the East African region at large. In fact, 75% of the households in Uganda are in the rural areas, hence further suggesting representativeness of the geographical scope in this study(33).

Additionally, the CSC processes involves a large number of activities and so accurately attributing the costs and cost savings is always a challenge. However, it's important to note that resource optimization and economies of scale can be attained as the number of sub counties covered by the CSC increase, although that would require a higher level of organization and coordination. Whereas the societal perspective is widely considered as being the most comprehensive to reflect the decision maker's perspective(34, 35), our cost analysis focused on only the provider's perspective. Although this could affect generalizability of the results and the overall estimated costs of scale up, we think that the effect is likely to be minimal since the additional opportunity costs (that would have been captured as part of the computed societal costs) are likely to be minimal due to the low earnings of these rural populations. That notwithstanding, the societal perspective not only focuses on broad cost aspects of the society's total welfare, but also allows capturing of the value of all changes in resources used as a consequence of a given intervention(s) (36).

The implementation period for the study of one year was also too short for us to comprehensively capture the actual scale-up costs. To address this gap, we used simulations to estimate the CSC scale-up costs within the entire Kibuku district. Future studies could do simulations that reflect the nation-wide cost of CSC implementation putting in consideration several key factors such as the size of different districts (in terms of geography and population), availability of district staff to implement/supervise the CSC activities, existence of and partnerships with civil society, degree of urbanization and variation in socio-economic status. Future research could also consider conducting a comprehensive economic evaluation to estimate the net cost implications for CSC implementation in low- and middle-income countries, so as to inform ongoing efforts to incorporate CSC and other Social Accountability mechanisms into health programming. These will ultimately support financing for CSC to foster improvements in quality of maternal and newborn health services as well as outcomes in resource limited settings like Uganda.

Conclusion

Our paper is one of the few that attempt to estimate the cost of implementing CSC implementation in resource limited settings. Furthermore, our analysis has also provided simulations for costs that may be required to implement a CSC at scale, at the different levels of administration or service delivery – the sub-county and the district. The overall cost of implementing the CSC intervention in five subcounties and one town council in Kibuku district was USD 59,962. The average cost of implementing the CSC per scoring round per sub-county was USD 1,998. The main cost drivers were transportation costs, coordination and supervision costs to support the technical capacity of the local implementers. The other cost drivers included transport refunds and provision of refreshments and meals for the dissemination and monitoring and evaluation meetings.

Our analysis suggests that it is financially feasible to implement and scale-up the CSC initiative, as an accountability tool for enhancing service delivery. However, the CSC approach and implementation design must be one that is contextual, optimizes costs, including ensuring adequate involvement of local communities and stakeholders. Under the scale-up simulations, scenario two (considering cost inputs from the district implementation teams) was noted to have the lowest CSC implementation costs with an estimated annual CSC implementation cost of USD 28,465 across the entire district (22 subcounties). There are opportunities to finance CSC activities within the available funding streams; for example, leveraging Sub-county and district budgets to support operational costs and the existing political will to enhance accountability through the constituency assembly meetings.

Abbreviations

DFID: Department for International Development; FHS: Future Health Systems Research Program Consortium; MakSPH: Makerere University School of Public Health; CSC: Community Scorecard; MNH: Maternal and Newborn Health; VHT: Village Health Teams; DHT: District Health Team; WHO: DHO: District Health Office(r); World Health Organization

Declarations

Ethics approval and consent to participate

Ethical approval was obtained from the Makerere University School of Public Health Higher Degrees Research and Ethics Committee (MakSPH HDREC) and the Uganda National Council of Science and Technology (UNCST), study number SS 4323.

Consent for publication

Not applicable.

Availability of data and materials

Data sharing is not applicable to this article as no datasets were generated or analyzed during the current study.

Competing interests

The authors declare that they have no competing interests.

Funding

The project was supported by DFID through the Future Health Systems (FHS) Consortium program under the Award Number HRPC09 Delivering Effective Health Services. The content of this manuscript is solely the responsibility of the authors and does not necessarily represent the official views of the DFID. We also acknowledge the Kibuku district administration, the research assistants and our study participants.

Authors’ contributions

All authors contributed to the collation of documents and data used to inform this paper. AS drafted the initial manuscript based on the inputs received from the author team. LP and EEK provided overall guidance and support for the paper, as well as detailed comments on all drafts. EBN, RRA and EEK participated in scoring meetings within the implementation district, contributed to the collation of literature and project documents and provided edits and comments on all drafts. EBN, RRA And EEK did the analysis for the paper. All authors read and approved the final version of the manuscript.

Acknowledgements

The authors would like to acknowledge the helpful review and feedback provided by Chrispus Mayora, Ligia Paina, Natalia Alfonso during the development of this manuscript. We also acknowledge the support provided by Christine Aanyu in identification of data inputs used in this study.

References

1. Ho LS, Labrecque G, Batonon I, Salsi V, Ratnayake R. Effects of a community scorecard on improving the local health system in Eastern Democratic Republic of Congo: qualitative evidence using the most significant change technique. Conflict and health. 2015;9(1):27.

2. George A. Using accountability to improve reproductive health care. Reproductive health matters. 2003;11(21):161-70.

3. Mafuta EM, Dieleman MA, Hogema LM, Khomba PN, Zioko FM, Kayembe PK, et al. Social accountability for maternal health services in Muanda and Bolenge Health Zones, Democratic Republic of Congo: a situation analysis. BMC health services research. 2015;15(1):514.

4. Mutale W, Stringer J, Chintu N, Chilengi R, Mwanamwenge MT, Kasese N, et al. Application of balanced scorecard in the evaluation of a complex health system intervention: 12 months post intervention findings from the BHOMA intervention: a cluster randomised trial in Zambia. PLoS One. 2014;9(4):e93977.

5. Blake C, Annorbah-Sarpei NA, Bailey C, Ismaila Y, Deganus S, Bosomprah S, et al. Scorecards and social accountability for improved maternal and newborn health services: A pilot in the Ashanti and Volta regions of Ghana. International Journal of Gynecology & Obstetrics. 2016;135(3):372-9.

6. Edward A, Osei-Bonsu K, Branchini C, shah Yarghal T, Arwal SH, Naeem AJ. Enhancing governance and health system accountability for people centered healthcare: an exploratory study of community scorecards in Afghanistan. BMC health services research. 2015;15(1):299.

7. Osrin D, Das S, Bapat U, Alcock GA, Joshi W, More NS. A rapid assessment scorecard to identify informal settlements at higher maternal and child health risk in Mumbai. Journal of Urban Health. 2011;88(5):919-32.

8. Yilla M, Nam SL, Adeyemo A, Kargbo SA. Using scorecards to achieve facility improvements for maternal and newborn health. International Journal of Gynecology & Obstetrics. 2014;127(1):108-12.

9. Gullo S, Galavotti C, Altman L. A review of CARE's Community Score Card experience and evidence. Health Policy and Planning. 2016;31(10):1467-78.

10. Joshi A. Do they work? Assessing the impact of transparency and accountability initiatives in service delivery. Development Policy Review. 2013;31:s29-s48.

11. Martin Hilber A, Blake C, Bohle LF, Bandali S, Agbon E, Hulton L. Strengthening accountability for improved maternal and newborn health: A mapping of studies in Sub- Saharan Africa. International Journal of Gynecology & Obstetrics. 2016;135(3):345-57.

12. Bennett S, Mahmood SS, Edward A, Tetui M, Ekirapa-Kiracho E. Strengthening scaling up through learning from implementation: comparing experiences from Afghanistan, Bangladesh and Uganda. Health research policy and systems. 2017;15(2):108.

13. Galavotti C, Gullo S, Altman L. A review of CARE's Community Score Card experience and evidence. Health Policy and Planning. 2016;31(10):1467-78.

14. Misra V. Pilot Study 1, Andhra Pradesh, India: Improving Health Services through Community Scorecards. Learning Notes, Social Accountability Series. 2007.

15. Gaventa J, McGee R. The impact of transparency and accountability initiatives. Development Policy Review. 2013;31:s3-s28.

16. Björkman M, Svensson J. Power to the people: evidence from a randomized field experiment on community-based monitoring in Uganda. The Quarterly Journal of Economics. 2009;124(2):735-69.

17. Ekirapa-Kiracho E, Apolot R, Kiwanuka S. Which contextual factors facilitate successful implementation of Community Score Cards in Uganda? 2018.

18. UNICEF. Annual Results Report 2017. New York, USA: United Nations Children's Fund; 2018.

19. Ministry of Health. Implementation Guide for Uganda's Reproductive Maternal, Newborn and Child Health Balanced Score Card. Kampala, Republic of Uganda.2015.

20. UBOS. Uganda Subcounties 2014. In: Uganda Bureau of Statistcs, editor. Dataset ed. Kampala, Uganda2014.

21. Katende D, Mutungi G, Baisley K, Biraro S, Ikoona E, Peck R, et al. Readiness of Ugandan health services for the management of outpatients with chronic diseases. Tropical medicine & international health. 2015;20(10):1385-95.

22. Müller- Nordhorn J, Brüggenjürgen B, Böhmig M, Selim D, Reich A, Noesselt L, et al. Direct and indirect costs in a prospective cohort of patients with pancreatic cancer. Alimentary pharmacology & therapeutics. 2005;22(5):405-15.

23. Elizabeth Ekirapa-Kiracho, Christine Aanyu, Rebecca Racheal Apolot, Ligia Paina, Sara Bennett, Kiwanuka. SN. Designing for Scale and Taking Scale to Account: Lessons from a community score card project in Uganda (Upcoming). International Journal for Equity in Health. 2019.

24. Walker D. Cost and cost-effectiveness guidelines: which ones to use? Health Policy and Planning. 2001;16(1):113-21.

25. Cunnama L, Sinanovic E, Ramma L, Foster N, Berrie L, Stevens W, et al. Using top- down and bottom- up costing approaches in LMICs: The case for using both to assess the incremental costs of new technologies at scale. Health economics. 2016;25:53-66.

26. Levin HM. Cost-effectiveness: A primer: Sage Publications, Inc; 1983.

27. Levin HM, Belfield C. Guiding the development and use of cost-effectiveness analysis in education. Journal of Research on Educational Effectiveness. 2015;8(3):400-18.

28. Razavi SD, Kapiriri L, Abelson J, Wilson M. Who is in and who is out? A qualitative analysis of stakeholder participation in priority setting for health in three districts in Uganda. Health policy and planning. 2019.

29. Patel Darshana, Shah Parmesh, Moutushi. I. Impact of Social Accountability Mechanisms on Achieving Service Delivery and Health Development Outcomes in Satara District, Maharashtra, India. Social Accountability Series: South Asia Suistainable Development Department; 2009.

30. Kabunga NS, Mogues T, Bizimungu E, Erman A, Van Campenhout B. The State of Public Service Delivery in Uganda: Report of a Baseline Survey: Intl Food Policy Res Inst; 2016.

31. Howard J, López Franco E, Shaw J. Navigating the Pathways from Exclusion to Accountability: From Understanding Intersecting Inequalities to Building Accountable Relationships. 2018.

32. Post D, Agarwal S, Venugopal V. Rapid feedback: the role of community scorecards in improving service delivery. 2014.

33. UBOS. The National Population and Housing Census 2014 – Main Report. Kampala, Uganda: Uganda Bureau of Statistics; 2016.

34. Sanders GD, Neumann PJ, Basu A, Brock DW, Feeny D, Krahn M, et al. Recommendations for conduct, methodological practices, and reporting of cost-effectiveness analyses: second panel on cost-effectiveness in health and medicine. Jama. 2016;316(10):1093-103.

35. Ismail A, Suddin LS, Sulong S, Ahmed Z, Kamaruddin NA, Sukor N. Economic burden of managing Type 2 diabetes mellitus: Analysis from a Teaching Hospital in Malaysia. Indian journal of public health. 2017;61(4):243.

36. Huter K, Kocot E, Kissimova-Skarbek K, Dubas-Jakóbczyk K, Rothgang H. Economic evaluation of health promotion for older people-methodological problems and challenges. BMC health services research. 2016;16(5):328.

Tables

Table 1: Key Community Scorecard Implementation Processes

Process	Elaboration of the process
**Preparatory ground work (planning meetings, Community mobilization and sensitization and Input tracking matrix preparation)**	Planning meetings. This involved meetings at district and sub-county levels with the political, technical, community leaders, health facility management and other stakeholders to solicit buy-in for the CSC project and select intervention locations. It also involved development of training materials and training of the CSC facilitators and coordinators.
	Community mobilization and sensitization. Through the district and sub-county political and technical leadership, the participants for the community scoring were identified and mobilized. Radio spot messages were also aired and patient charters printed and pinned up in different locations within the intervention area to sensitize the different stakeholders of their roles and responsibilities in MNH.
	Input tracking matrix preparation. The health workers, District Health Team and technical support team from MakSPH conducted in-put tracking by looking at the standards required for different health facility levels vis-à-vis what was available on ground. They tracked; infrastructure, equipment, and staffing. This tracking was conducted once at the beginning of the project.
**Health facility Scoring**	During the facility scoring, the health workers of the 5 selected health facilities listed their priority MNH indicators; scored them using colors and developed a facility score card and a work plan for improvement. These were later presented in the interface meetings. This was repeated quarterly.
**Community scoring**	Community scoring was done through 20 FGDs (10-female, 10-male) with selected community members from all parishes in six sub counties in the intervention area. Each group comprised of 12 FGD participants. In the first community score card meeting (round 1) they identified and prioritized the indicators and then they scored the indicators using colours and developed work plans for improvement. In the subsequent rounds 2 and 3 they met and scored the indicators. In rounds 4 and 5, the scoring was done during the interface meeting that happened at parish level.
**District scoring**	The ***district scoring*** session was attended by 20 participants who discussed district performance as per the quarterly RMNCAH score card generated by MoH and developed a work plan for improvement. The DHT members also discussed MNH issues that had been raised by the communities during the scoring sessions.

**Interface meetings**	In ***ROUND 1 of scoring***, five interface meetings were held at sub-county level. During scoring rounds; 2, 3, 4 and 5, interface meetings were held at parish level totalling to 25 meetings in order to strengthen participation of community members. Participants at interface meetings included community members, health workers, DHT members, political and technical leaders, and other stakeholders.<br>During the ***ROUND 3 of scoring,*** local council members started doing the mobilization for the interface meetings in an attempt to improve attendance and participation of community members.
**Dissemination**	After each scoring round, stakeholders were briefed about findings from different sub-county and district scorecards and workplans. The stakeholders included; district and sub-county political and technical leaders, health workers, civil society organisations (CSOs), community leaders, and Health Unit Management Committee (HUMCs).
**Monitoring and evaluation**	The monitoring and evaluation activities comprised of feedback meetings and follow up meetings. After each scoring round, feedback meetings were arranged as a forum for facilitators from all the sub-counties to meet and discuss their scores and work plans. They also discussed, the gaps and challenges that had been identified and proposals to address them going forward. DHT members and the Makerere University School of Public Health (MakSPH) research team guided the feedback meetings. Fifteen (15) follow up meetings (1per Sub-county per scoring round for rounds 1, 2 and 3) were carried out with different sub-county councils and stakeholders who were responsible for oversight of implementation of agreed upon activities in the CSC work plans for their sub counties.

Table 2: CSC Costing Categories for the Startup and Operational Costs

Cost category	Description of the category
**Training**	Initial training costs included costs related to training of DHTs*, implementation teams, HUMCs** and VHTs***.<br>Refresher training costs included transport refund and refreshments provided to the participants during the refresher training that occurred before each round of scoring.
**Mobilization and sensitization**	Costs related to mobilization and sensitization meetings held at district sub-county, parish, and community levels at the start of the project. Additional costs were incurred on media engagements including talk-shows and radio spots, and design and printing of the patient charter. During the implementation mobilization was

	done using a public address system and by the local council members in round 2,3 and 4.
**Coordination costs**	These captured costs incurred on allowances for core personnel for MakSPH (50% of the time for the CSC coordinator) involved in initial planning and design, and allowances for coordinators from the district and sub-county.<br>Other costs here included airtime for coordination of scoring activities.
**Supervision costs**	This category captured costs incurred on core personnel involved in providing technical support and supervision from MakSPH and the District Health Team. Other costs here included transport for regular monitoring, supervision.
**Transport**	Transport costs included all costs spent on transportation requirements
**Facilitation costs**	Facilitation costs included all payments made to the facilitators of the CSC meetings
**Refreshments**	Costs on refreshments included all the costs incurred to provide meals and refreshments during the meetings
**Monitoring and evaluation costs Feedback meetings and sessions**	These costs included costs for the feedback and follow up meetings. The Costs incurred during these sessions included: transport refunds or allowances for participants, and refreshments.
**Dissemination meetings.**	This comprised of costs incurred during the quarterly stakeholder meeting; refreshments and transport refund for different stakeholders, coordinators and DHT for all the five scoring rounds. Each dissemination meeting had an average of 60 participants.
**Stationery**	This included all costs spent on stationery used during the CSC process

592 **DHT-District Health Team: **HUMCs-Health Unit Management Committees:*

593 ****VHTs-Village Health Team*

594

595

596 **Table 3: Parameters Used for Simulation of Community Scorecard Scale-up Scenarios**

**Cost inputs**	**Costs included in scenario one**	**Costs included in scenario two**	**Justification for inclusion**
**Initial training**	√	√	Initial training will be required to impart the necessary skills in both scenario 1and 2
**Community mobilization**	√		Community mobilization and sensitization will be required to equip the community with information about the CSC but in scenario 2,

			only existing meetings will be used and so no extra costs are included.
**Stakeholder meeting at subcounty level**	√		Stakeholder meetings will be required for buy in. In scenario 2 this will be done as part of another ongoing activity and so no extra costs are attached
**Mobilization**	√		Local council chair persons will mobilize participants for the interface meetings in scenario1, in scenario 2 they will do the mobilization without any payment
**Stationery**	√	√	Stationery will be required for the scoring activities in both scenarios
**Preparatory meeting at subcounty level**	√		Preparatory meetings will be held for half a day and will attract costs for break tea and safari day allowance. In scenario 2 the meetings will be much shorter about 1 hour and will not attract any financial costs.
**Health facility scoring**			Meetings will attract no additional costs in both scenarios since they will be done during routine facility review meetings
**Scoring and interface meetings**	√	√	Safari day allowance of USD 5 will be paid to facilitators in scenario 1 while only transport refund of USD 2.77 will be paid in scenario 2.
**Feedback meeting at subcounty**			Feedback will be done after each interface meeting so that no extra costs are incurred in both scenarios
**Stakeholder dissemination meeting**			Dissemination of CSC findings will be done during routine quarterly joint stakeholder meetings at the district and subcounty council meetings so that no extra costs are incurred in both scenarios
**Sub-county coordinator and Co-coordination costs**	√	√	The subcounty coordinators will receive a safari day allowance for their facilitation in both scenarios
**DHT members supervision costs**	√		The DHT members will receive a safari day allowance for their facilitation in scenario 1 while in scenario 2 supervision will be done by the Subcounty coordinators

Table 4: Summary of Costs for implementation of the Community Scorecard Processes

Activities	Startup costs	Round one	Round two	Round three	Round four	Round five	Total	%allo cation
**Preparatory groundwork**	11413						11413	19%
Operational costs								
**Health facility scoring**		683	911	851	1189	917	4550	8%
**Community scoring**		1245	1473	1410	956	843	5926	10%
**District scoring**		663	660	781	1138	813	4055	7%
**Interface meetings**		2140	2138	2804	2404	2231	11717	20%
**Dissemination**		1488	1729	1704	2247	1870	9036	15%
**Monitoring and Evaluation**		3065	2960	2702	2669	1869	13265	22%
**Total**	**11413**	**9283**	**9870**	**10252**	**10602**	**8542**	**59962**	**100%**

Table 5: CSC Implementation Costs (USD)

Cost inputs	Startup Costs	Round one	Round two	Round three	Round four	Round five	Total	% allocat ion
**Coordination**	1333	1014	1409	1820	2167	1778	9521	16%
**Supervision**	1055	1333	2333	1607	2417	1597	10342	17%
**Training**	4300	-	-	-	-	-	4300	7%
**Mobilization**	1722	712	671	756	765	765	5391	9%
**Transportation**	1763	4364	3634	4043	3566	2683	20053	33%
**Facilitation**	806	853	804	992	805	954	5213	9%
**Refreshments**	376	757	768	818	739	625	4084	7%
**Stationery**	57	250	250	217	144	139	1058	2%
**Total**	**11413**	**9283**	**9870**	**10252**	**10603**	**8541**	**59962**	**100%**

Table 6: Costs of Community Scorecard Scale-up in Kibuku district by scenario

**Cost inputs**	Scenario one				Scenario two			
	**Single round cost (USD)**		**Annual cost (USD)**		**Single round cost (USD)**		**Annual cost (USD)**	
	Sub county*	District	Sub county	District	Sub county*	District	Sub county	District

Startup costs								
**Initial training**	949	6432	3794	25728	949	5821	3794	23283
**Community mobilization**	270	1625	6500	6500	-	-	-	-
**Stakeholder meeting (sub-county level)**	311	6844	1244	27378	-	-	-	-
**Stationery**	12	264	48	1056	6	244	22	978
Sub-total - startup costs	*1542*	*15165*	*11587*	*60661*	*954*	*6065*	*3817*	*24261*
Operational costs								
**Mobilization**	25	550	100	2200	-	-	-	-
**Preparatory meeting at sub-county level**	64	1406	256	5622	-	-	-	-
**Scoring and interface meetings (per diem for facilitators)**	40	880	160	3520	22	489	89	1956
**District coordination and sub-county coordination**	20	440	80	1760	20	440	80	1760

**allowance**								
**Stationery**	6	122	22	489	6	122	22	489
**DHT members supervision costs**	20	440	80	1760	-	-	-	-
Sub-total - Operational costs	*175*	*3838*	*698*	*15351*	*48*	*1051*	*191*	*4205*
**Grand total**	**1716**	**19003**	**12285**	**76012**	**1002**	**7116**	**4008**	**28465**

**Average Costs reported per sub-county* Annual *Costs reported for CSC implementation reported over for quarters*

Click here to access/download

Supplementary Material

SUPPLEMENTARY FILES-vrsn 1.docx
