# Supplementary material for: Influence of community scorecards on maternal and newborn health service delivery and utilization
Source: Int J Equity Health. 2020 Nov 2;19:145. doi: 10.1186/s12939-020-01184-6 (PMC7604954; doi:10.1186/s12939-020-01184-6)
Supplement: Supplementary file 2 — Additional file 2.Designing for Scale and Taking Scale to Account: Lessons from a community score card project in Uganda. [file 12939_2020_1184_MOESM2_ESM.pdf]

Designing for Scale and Taking Scale to Account: Lessons from a community score card project in Uganda

Elizabeth Ekirapa Kiracho[1], Christine Aanyu[1], Rebecca Racheal Apolot[1], Sara Bennett[2], Suzanne N. Kiwanuka[1], Ligia Paina[2]

[1] Makerere University School of Public Health

[2] Johns Hopkins University Bloomberg School of Public Health

Corresponding author

Christine Aanyu; Department of Health Policy Planning and Management, Makerere University School of Public Health, P.O.Box 7072, Kampala, Uganda. (aanyuchristinee@gmail.com/caanyu@musph.ac.ug)

Elizabeth Ekirapa Kiracho, Department of Health Policy Planning and Management, Makerere University School of Public Health, P.O. Box 7072, Kampala, Uganda. ekky@musph.ac.ug / ekky01@gmail.com

Rebecca Racheal Apolot, Department of Health Policy Planning and Management, Makerere University School of Public Health, P.O. Box 7072, Kampala, Uganda. apobecca@gmail.com/apobecca@musph.ac.ug

Ligia Paina; Department of International Health, Johns Hopkins Bloomberg School of Public Health, 615 N. Wolfe Street, Baltimore, MD 21205, United States of America. lpaina@jhu.edu

Sara Bennett, Department of International Health, Johns Hopkins Bloomberg School of Public Health, 615 N. Wolfe Street, Baltimore, MD 21205, United States of America. sbennett@jhu.edu

Suzanne Namusoke Kiwanuka, Department of Health Policy Planning and Management, Makerere University School of Public Health, P.O.Box 7072, Kampala, Uganda. skiwanuka@musph.ac.ug

Abstract

Background

Planning for the implementation of community scorecards (CSC) is an important, though seldom documented process. Makerere University School of Public Health (MakSPH) and Future Health Systems Consortium set out to develop and test a sustainable and scalable CSC model. This paper documents the process of planning and adapting the design of the CSC, incorporating key domains of the scalable model such as embeddedness, legitimacy, feasibility and ownership, challenges encountered in this process and how they were mitigated.

Methods

The CSC intervention comprised of five rounds of scoring in five sub counties and one town council of Kibuku district. Data was drawn from ten focus group discussions, seven key informant interviews with local and sub national leaders, and one reflection meeting with the project team from MakSPH. More data was abstracted from notes of six quarterly stakeholder meetings and six quarterly project meetings. Data was analyzed using a thematic approach, drawing constructs outlined in the project's theory of change.

Results

Embeddedness, legitimacy and ownership were promoted through aligning the model with existing processes and systems as well as the meaningful and strategic involvement of stakeholders and leaders at local and sub national level. The challenges encountered included limited technical capacity of stakeholders facilitating the CSC, poor functionality of existing community engagement platforms, and difficulty in promoting community participation without

financial incentives. However, these challenges were mitigated through adjustments to the intervention design based on the feedback received.

Conclusion

Governments seeking to scale up CSCs and to take scale to account should keenly adapt existing models to the local implementation context with strategic and meaningful involvement of key legitimate local and sub national leaders in decision making during the design and implementation process. Social accountability practitioners should document their planning and adaptive design efforts to share good practices and lessons learned. Enhancing local capacity to implement CSCs should be ensured through use of existing local structures and provision of technical support by external or local partners familiar with the skill until the local partners are competent enough to conduct CSC activities, including facilitation, negotiation, mediation and community mobilization.

Key words: Community Score Cards, scale up, accountability, theory of change

Introduction

For decades governments in low income countries have failed to provide poor populations with adequate social services to meet their needs (1,2). Social accountability is increasingly being seen as an approach that could augment public sector actions to meet the needs of the poor (2). The community score card (CSC) is one of the social accountability tools that has been employed to monitor the availability, access and quality of social services (3,4). Use of CSCs has contributed to increased expression of community and health provider concerns, improved responsiveness and accessibility of health services in addition to improved accountability, quality as well as improved communication between service providers and users (4–9). However, evidence regarding the effect of prevailing social accountability tools is mixed, with some authors reporting enhanced accountability and others reporting the opposite (1,10–13). The CSCs picture in Uganda is similar to the global picture, where community score cards have been implemented largely as pilot projects without national scale up (14).

Scale-up is commonly defined as efforts to increase the impact of the innovations successfully tested in pilots or experimental projects so as to benefit more people and foster policy and programme development on a lasting basis (15). Scalability is defined by Milat et al., 2012 as the ability of a health intervention shown to be efficacious on a small scale and or under controlled conditions to be expanded under real world conditions to reach a greater proportion of the eligible population, while retaining effectiveness (16). However, the accountability literature argues that simply increasing or expanding the scale of doing something may not necessarily achieve the desired objective of increasing accountability (17). Increasing accountability requires that specific action is taken to address the underlying accountability failures through upward

vertical integration between various actors at local, sub national and national levels so as to get more leverage over more powerful institutions. This is what Fox refers to as "*taking scale into account*" (17). "Taking scale into account" then is less about scaling up to more locations/geographies, and more about working through and across different levels of decision-making and practice from local to sub national to national (11,17). It should begin in the initial planning and design phases, but can only happen if practitioners look beyond the details of the specific intervention that they are piloting into the broader enabling environment. This facilitates coordinated action among different actors that allow horizontal and vertical coalitions to develop and bring about desired actions that transform the behavior of health system actors so as to promote a culture of accountability and accountability systems (17,18) (19).

With growing interest in strengthening social accountability to make progress towards Universal Health Coverage, the issues of scaling up and institutionalizing promising pilot projects, is therefore timely. If we assume that the factors influencing scale-up are dependent on the initial planning and design of the intervention, documenting this process, as well as how the design adapts over time relative to what it is trying to achieve is important, though seldom done.

The theory of change in this paper serves as a reference point for iterative and adaptive design during implementation (20). It provides a framework for the analysis of feasibility, embeddedness, ownership and legitimacy and also helps to enrich the understanding about the pathways of change, as outlined by Wild and Harris (21) and documented by Ekirapa-Kiracho et al (22).

The purpose of this paper is to document the iterative design and planning undertaken by the MakSPH team to support the implementation of a community score card pilot in five sub

counties and one town council in Kibuku district, located in the Eastern region of Uganda with a population of 202,033 people (23).

Methods

The CSC intervention was conducted for five quarterly rounds between the months of June 2017 to December 2018. The CSC intervention consisted of eight main stages; as illustrated in Figure 1, below (24). More details about the CSC intervention can be obtained from the paper by Ssebagereka et al and Ekirapa-Kiracho et al (22,25).

Figure 1 here

Theory of change for CSC implementation

The planning phase was guided by a theory of change (see figure 2), whose development was guided by previously published scaling up frameworks (15,16,26–29), in particular the Expand Net framework (30), the FHS project institutionalization framework (31) as well as project wide discussions.

Figure 2 here

According to our theory of change, four main factors were central to ensuring that the CSC designed was scalable and sustainable. These included embeddedness (entrenchment into already existing systems or processes or policies at the local or national level), legitimacy (working with persons/structures that are mandated to carry out specific activities), feasibility (low cost, simplicity of tools, acceptability, less human resource intensive) and ownership (high level

stakeholder participation and acceptance of the CSC). We believed that the inclusion of these components would facilitate the ability of the CSC to stimulate collective action by the community, health providers, health facility managers, sub county and the district leaders. These actions would then act through the six pathways proposed by Wild and Harris to bring about the desired changes at various levels (21). The six pathways include strengthening citizens' demand, increased resourcing, improving information flows, greater top down performance pressure, collective action on the side of citizens and collective action encompassing demand and supply (21). If these actions addressed the needs of the community and the leaders then we believed that the chances of institutionalizing, sustaining and taking scale into account of the CSC would be increased.

Our theory of change had five main assumptions; firstly, if the CSC was embedded in existing structures, it had a greater chance of institutionalization and sustainability. Working at community, health facility, sub county, district and national levels would increase buy in and influence decision making processes to favor the needs of the community members. Secondly, CSCs that use legitimate stakeholders would trigger collective action from communities, providers and district officials. Thirdly, if there was ownership by various levels of stakeholders, it would be easy to sustain, institutionalize and scale-up the CSC. Fourthly, if the CSC was feasible then it would most likely be sustained, institutionalized and scaled-up and lastly if we took scale into account, then the CSC could be sustained beyond the life of the project.

Data collection methods

We conducted ten focus group discussions (FGDs); five female and five male and seven key informant interviews (KIIs) as well as one reflection meeting with the project team. Data was

also abstracted from quarterly project and stakeholder meeting reports.

All FGDs and four KIIs were conducted in June 2018, while three KIIs were conducted in November 2018. KIIs were conducted with purposively selected technical and political leaders involved in the implementation of the CSC.

The 10 FGDs were randomly selected from the 20 FGDs involved in the first three rounds of scoring. Each FGD had 10-12 participants representing different categories of interest groups (women and men of reproductive age, disabled persons, people with different socio economic status, elderly) and the villages in that particular sub county.

The FGD and KII guides contained questions aimed at gathering information related to changes observed, facilitators, challenges/barriers, feasibility, sustainability, institutionalization and scaling up of CSC. FGDs and KIIs were conducted by trained research assistants fluent in both English and Lugwere (local language).

The reflection meeting was conducted once at the end of the fifth round of scoring with researchers from MakSPH. It was guided by a tool adapted from the ExpandNet 20 questions for developing a case study for scaling up (32).

Data was also abstracted from notes from the stakeholder and project meeting reports. These meetings were held with stakeholders from the district and sub county, implementers of the project as well as the research team from MakSPH every quarter throughout the 18 months' period of the project. High-level district political and technical leaders facilitated the stakeholders and project meetings.

Data management and analysis

All FGDs and KIIs were transcribed verbatim. During the stakeholder meetings, notes were taken and then later typed. All transcripts were read several times to allow familiarization with the data. We then developed an analytical framework based on key themes; embeddedness, legitimacy, feasibility and ownership guided by the ExpandNet framework as highlighted in the project theory of change. Codes were then developed and applied according to the analytical framework. Any new emerging codes related to the study objectives were also included (33).

Results

We present the actions that were taken to design and set up a scalable CSC model that takes scale into account by putting in place features that enhance ownership, embeddedness, legitimacy and feasibility of the CSC. We also present the challenges that were encountered and how they were mitigated.

Ownership of CSC

To promote ownership of the CSC process two main actions were taken by the MakSPH team, firstly a wide range of leaders from different levels at the district were engaged throughout the planning, design and implementation process. Secondly a participatory implementation design was used where modifications were made based on feedback from the facilitators of CSCs. The inclusion of political and technical leaders in the community, sub county and district levels, as well as fostering spaces for joint dialogue across these groups was important for securing buy-in and enhancing inclusion of locally appropriate plans based on their needs. Some of these leaders also participated as facilitators of the CSC meetings. This not only promoted buy-in, involvement and ownership of the intervention in all the sub counties, but also enhanced the implementation of the project as noted below.

"Chairpersons, Local council leaders (LC1s, LCVs) have also helped the score card and even the councilors have helped because whenever a person calls them whether they are in problems or in joy they hint on it [talk about the score card project] so in one way or the other they have helped the project to spread." Participant FGD Men sub county E.

"Also our leaders in the community like the LCs accepted the CSC to be implemented in the community that is one of the facilitators [for successful implementation] because if they [leaders] had refused the CSC team to implement these activities in the community they [leaders] would have given excuses like we [leaders] don't want to disturb our women …. But community leaders accepted their women to participate and not only women but even they themselves became part of this program because they started participating that is why it has been successfull." Participant FGD Women sub county B.

The participatory implementation design further promoted stakeholder buy-in, ownership and involvement of the local stakeholders**.** Local leaders at various levels with the mandate to mobilize and call for community meetings were involved to secure community buy-in. Feedback from facilitators was sought after every scoring to help identify what worked well and what did not during the implementation. This helped the MakSPH team modify the CSC process so as to make it more acceptable and enhance its chances for institutionalization and scale-up. For example, in the initial rounds of scoring, the process was reported to be extremely labor intensive and so in the last two rounds of scoring (fourth and fifth rounds), the MakSPH team combined the FGD and interface meetings into one community meeting held at parish level to reduce the human resource obligations which had previously made CSC labor intensive. Taking community feedback into account when re-designing the CSC intervention also helped to promote ownership as echoed below by one of the MakSPH team members:

"…other scorecards do not report anything about people in the field giving feedback and modifying the tool or modifying the process but we designed ours with feedback meetings where people were telling us about the challenges [encountered during scoring].We [MakSPH team]then modified our plans because we [MakSPH team] know that we [MakSPH team] are able to learn from that…..and this can be helpful especially for sustainability and future scale up and institutionalization because when it comes from the people, they are willing to take it up as a routine." MakSPH staff team 3

Embeddedness of the CSC

Embeddedness into the local structures and systems was promoted by working with political and technical officers from Kibuku district local government (user organization) and alignment with existing structures and policies. In the selection of user organizations, a choice had to be made between using locally based Non-Government Organizations (NGOs) working in a related accountability area and using other locally existing structures. However, in Kibuku district there was only one active NGO doing accountability related work, with minimal staff. Moreover, conducting CSC meetings was not one of the major activities in their work plan. During the design phase, stakeholders and the research team therefore decided to use multiple existing technical and political structures such as Senior Assistant Secretary (SAS), health unit management committees (HUMCs), Local council (LC) leaders and Village health team (VHT) members among others. These personnel existed in adequate numbers, and could carry out CSC activities as part of their daily activities since this was not outside their job description.

“The project found me [elected sub county political leader] when I was good at passing educative and helpful information to the community especially in the area of health and security so when I [elected sub county political leader] grasped the idea of the community score card, I [elected sub county political leader] integrated it with the previous programmes and whenever there are public gatherings [weddings, funerals, places of worship], I [elected sub county political leader] make sure I [elected sub county political leader] pass the information to them[community members]” KI Elected political leader sub county D.

“I think there are tradeoffs, ... you [project implementer] trade off the costs of having an independent NGO to run this and then eventually you cannot afford to pay them or you can’t sustain it or scale it up because you will not have an NGO all over the country and the tradeoff of having it be [very effective] so ... I think that it is all about strengthening the system enough to highlight the problems because eventually even the sub county chief [SAS] whom they may not be able to hold accountable, gets accountable if the spotlight gets on them because these communities I have seen are vocal, all they need is a platform, they will talk and the things will get recorded…” MakSPH Staff 2

However, the technical capacity of CSC facilitators from the existing structures was not optimal in some cases. An initial training was conducted over a five-day period for the core implementation team by MakSPH and the district health team (DHT). Thereafter their ability to facilitate a CSC meeting was assessed and those who were deemed too inept to carry out the required tasks were excluded. This was echoed by one of the MakSPH researchers.

“I think that working with the local facilitators, yes it has worked in terms of [reducing expenses] it is not very expensive because they come from within the communities but it

takes a lot of effort for capacity building... the people who are available in the communities and acceptable to the communities to do this kind of work and also who are willing to be volunteers in the community [may have a low level of education]" MakSPH staff 3.

Additionally, one day refresher training and technical support before each scoring round was provided on a quarterly basis by MakSPH and the DHT. The DHT, District Health Office (DHO) and the sub county technical and political leadership (SAS, Community Development Officers (CDOs), LC III chair persons and sub county councilors) also acquired skills for coordinating the CSC implementation process and took a key role in providing support as the implementation continued beyond the project life cycle.

*"...when you [MakSPH/resource team] were providing induction to us [facilitators and coordinators of the CSC], you [MakSPH team] gave us [facilitators and coordinators], enough time during the training so the facilitators understood what they were meant to do in the field during the scoring process...*KI Elected political leader district.

In order to promote embeddedness, MakSPH team aligned the CSC tools with other existing policy tools to avoid creation of duplicate tools or creation of parallel structures used by the NGOs when implementing CSCs as highlighted during the initial accountability mapping by the research team. For example during the facility scoring, poorly performing indicators from the

government led health facility Reproductive Maternal Newborn Child and Adolescent Health (RMNCAH) score card were also identified and targeted for action.

> "*I think one of the main things we [research team] did was that we co-created the whole idea, we did not come in with our own model(s), we did provide technical guidance on what needed to be done but the structures and the processes were informed by the people [local stakeholders] who were going to implement this. That means we identified the people mandated to do it,…platforms that were supposed to be used,… available tools and or the lack thereof and then we tried to strengthen both the human resource, the platforms and the structures so that whatever we did in terms of timing of these activities is primarily informed by the actual people on the ground who are mandated to do this work.*" MakSPH staff 2

However, getting entrenched into existing systems and processes requires adequate time and in some cases negotiation with key players. Although we tried to embed feedback meetings into existing platforms this was not always successful. Some of the platforms were nonfunctional for example some council meetings did not happen when there were no allowances for the councilors.

Legitimacy of the CSC

As noted above legitimacy was ensured by aligning CSC implementation within existing systems, policies and processes including; working with personnel who had the mandate to perform different tasks within the CSC process. This was considered important because such structures could potentially continue performing the expected services even after the project exits or continue with minimal additional pay since they (the local personnel) would be performing

duties that are within their mandate. These leaders felt that the CSC was enabling them fulfill their mandate and were therefore supportive of the programme and its continuity. In addition, they command the respect that is required from the community, as acknowledged in the quotation below.

> *"Yeah, we [sub county coordinators] involve them [political leaders] because when those people [political leaders] talk, people [community members] listen, when they [political leaders] say there is a meeting at a certain place there is a way people listen to the politicians more than the technical staff."* KI Technical leader sub county A.

Moreover, during the design phase, the technical and political leaders cautioned against designing a CSC which operates outside of the district system. They also noted that appointing district staff and assigning them roles outside their mandate results in officials overstepping their roles creating friction within the district.

Feasibility of CSC

The research team aimed at designing a simple low-cost intervention to enhance the feasibility for scale-up, sustainability, and institutionalization of the CSC. During the implementation of this intervention, several actions were undertaken to lower the associated costs. These included use of locally existing personnel who could be paid government allowance rates which are lower than rates often paid to NGOs, removal of refreshments for the community meetings and allowances for the community and health workers. These low cost implementation approaches were however not always welcomed by stakeholders who were used to receiving allowances from other projects and political leaders as noted in the quotations below.

"...the challenge has been that at the start when you [FGD participant] tell the person [community member], the person [community member] would just say aaha! what are they [CSC facilitators] going to give us [community members] and the person [community member] would say that for me, I can't go there [CSC meeting] where there is no "tea"[some transport refund/refreshment] I don't have time for you [FGD participant]." Participant, FGD Women, sub county A.

"The biggest challenge is one, we [district leaders] are aware that most of our people [community members] have been working when they are paid, so if they [funders] pulled out [withdrew funding] and if the district doesn't come in very fast with planning on how to integrate these activities [CSC activities] and leave it independent, we[district] may end up having a challenge because when you look around, most of our local technical staff here, they value money more than work...that is ...why we [district leaders] should start planning as we[resource team] are going to phase out..." KI Appointed political leader district.

However, the MakSPH team encouraged the community to look beyond the money and focus on the benefits like improvement in service delivery and utilization that they could get out of implementing CSCs beyond the project duration. The MakSPH team also encouraged them to identify alternative sources of funding including writing proposals to civil society organizations and budgeting for the CSC activities within the district and sub county budgets.

" ...we [MakSPH team] have also encouraged them [district political and technical leaders] to look into other opportunities for funding because we [MakSPH team] realized that much as we

want to reduce costs, there are costs which we [MakSPH team] cannot wish away; if someone [CSC facilitator/coordinator] needs transport to go to a meeting, they cannot walk to the meeting. They need transport so we [MakSPH team] have to see how they use their existing budgets or how to lobby certain partners to be able to meet these costs.” MakSPH staff 1.

The participatory design of implementation of CSCs was selected to allow flexibility during implementation. This enabled review of the implementation approach and simplification of aspects that were considered complex during each scoring round. This led to the modification of tools and meeting guides used during the initial scoring meetings making it easier for the facilitators to understand the tasks that they were required to carry out as they facilitated CSC meetings. The number of meetings was also reduced from 45 to 25 hence reducing workload on the facilitators and coordinators. The intervention was further simplified by transferring the responsibility of coordinating CSC meetings from two district coordinators to twelve sub county coordinators.

“…the intervention model, at first we [MakSPH team] had several meetings which was very hectic for people [community members and facilitators] so we felt that merging FGD scoring and interface meetings into one community scoring [meeting] made the intervention a little simpler… you [facilitator] did two things at ago instead of having separate meetings.” MakSPH Staff 3.

“…when we [facilitators] had just begun, they [community members] were complaining because remember we [facilitators] finished some of these [CSC] meetings at night but we [resource team/facilitators/coordinators] have tried to shorten our explanations, allowing us to go straight to the point.” KI Technical Respondent, sub county A.

However, some aspects of the intervention remained rather complex and could potentially have hindered scale up for example the initial process of selection of indicators. This activity was difficult for most of the participants and this could affect the potential for scale-up, sustainability and institutionalization of the CSC. Since it was done once, it was not possible to repeat this aspect of the intervention. Another activity that was done once and also noted to be rather complicated for some of the facilitators was the development of action plans.

Discussion

Whereas the scale up literature often puts emphasis on the ability to implement an intervention on a large geographical scale, taking scale into account for social accountability interventions emphasizes the importance of putting in place deliberate actions that encourage strategic partnerships that can enhance accountability by leveraging the influence of more powerful parties/stakeholders (17). In the discussion we reflect on the extent to which we were able to achieve both these aims by using a model that aimed at enhancing embeddedness, feasibility, ownership and legitimacy.

We found that by far the most important domains for enabling wide scale implementation within our framework were feasibility and ownership. To make the CSC feasible and scalable, attention should be paid to its design, technical capacity of implementers and the cost of implementation. The design should be simple without overly complicated processes and tools to allow stakeholders with limited capacity to use them (1). This calls for flexibility during implementation to allow modification of the model and its implementation (34). The complexity of the CSC process with regard to the number of meetings held and the time commitments for

both the community members who attend the meetings as well as the facilitators of these meetings also affected the feasibility of implementing the intervention on a wide scale. Reducing the number and length of meetings therefore redeemed time and simplified the CSC process making it more feasible to the implementing team and other stakeholders.

According to Ekirapa-Kiracho et al (14), one of the barriers to implementation of CSCs identified in earlier projects in Uganda, was the human resource intensity of the CSC process (14). In our CSC process, we made changes by reducing the number of meetings hence time commitments for both the implementers and community members. Additionally, the facilitators of CSCs should also have the technical capacity required to facilitate the CSC if it is to be implemented sustainably using existing structures (1,35). This was achieved by the training that was offered to the district stakeholders who acted as facilitators and coordinators during the CSC scoring. Selection criteria of the facilitators by the implementers should therefore ensure that their capacity to carry out the required tasks is included. If the local facilitators lack this capacity, a team external to the district should provide support with the aim of enabling the district to strengthen its own capacity to support CSC activities (14). Furthermore, existing teams need to be available in adequate numbers to carry out scoring as required (35). Whereas we did the scoring quarterly, implementers should consider bi annual scoring if it is to be done as a routine activity.

High costs were also noted as factors that constrained scale up of interventions including social accountability interventions (14). To keep costs low it is important to minimize the inclusion of inputs that may attract high costs. Further details about the cost of implementing CSCs can be obtained from Ssebagereka et al (25).

Information access and citizen voice are often not enough to deliver accountability (17,19,36,37). They need to be accompanied by the support of powerful leaders and building of relationships (1,17). Local ownership and legitimacy were therefore particularly pivotal for taking scale into account. Working with legitimate persons enabled us to involve leaders who had the authority and mandate to take the required actions at community and sub national levels. Leaders at different levels can play a critical role in influencing the scale of impact of the CSCs. While legitimate community level leaders can play an important role in ensuring that the CSC's are locally accepted and implemented successfully, they may not have much leverage in influencing upstream factors but can build coalitions with powerful stakeholders at higher levels. It is therefore important to plan for early and continuous meaningful engagement of leaders at community, district and national level (17,38). In Uganda community score cards are not routinely implemented under existing public sector processes. There are ongoing discussions with the national leadership to identify appropriate entry points for carrying out community score cards routinely and linking them with existing decision making platforms. This requires that the CSC processes are conducted by legitimate persons and embedded into the routine public sector processes aimed at enhancing accountability. Legitimacy and embeddedness are particularly important for scale up if the implementation model is relying on the use of existing public sector processes and systems.

Leaders also need to appreciate the benefits of their participation in the CSC to secure their buy-in and active participation in holding duty bearers accountable. It is therefore important to ensure that the CSC design allows the CSC to identify and contribute to meeting the local needs. From our findings, the key technical and political stakeholders and leaders interviewed reported that the CSC provides a useful method of assessing their performance giving them an opportunity to

identify and solve problems affecting their communities (35). Hence their desire and enthusiasm to see the CSC implementation continue on a wider scale. These kinds of interactions can also lead to a scale shift where you find a large scale change in accountability as a result of influence from specific key leaders (17). Such changes can then be embedded into local systems by their inclusion in work plans, budgets and job descriptions (38).

One of the shortcomings of using legitimate persons may be political and elite capture (39). If this is detected, steps should be taken to overcome it by leveraging the support of the pro accountability actors. Another challenge was frequent changes in leadership. For example, during the eighteen months' period of this pilot, two of the top technical and political leaders in the district were changed. Other authors have sighted this as a barrier to scaling up interventions (33).

Heavy reliance on interviews done among community members, leaders and the research team who were involved in the implementation of the project is one of the limitations of the study. This may have biased the responses. However, these interviews were triangulated by considering responses from all the different groups of stakeholders involved. Furthermore, we reported both positive and negative findings. Another limitation was the short implementation period which was inadequate for observing scale up. Furthermore, this design did not allow us to assess the extent to which the community voice was truly realized. We recommend this as an area for further research.

Conclusions

Embeddedness, legitimacy and ownership were mainly encouraged and promoted through alignment with existing processes and systems as well as meaningful and strategic involvement

of the stakeholders and local leaders at local and sub national level. The key factors that enhanced feasibility included use of a simple low cost design that was implemented by locally existing stakeholders. The use of a participatory implementation design with mechanisms for continuous support during implementation and availability of minimal funding for supporting key activities were also central to the success of the implementation process. Governments seeking to scale up CSCs and to take scale to account should keenly adapt existing models to the local implementation context with the strategic and meaningful involvement of legitimate key local and sub national leaders in decision making during the design and implementation process. Social accountability practitioners should document their planning and adaptive design efforts in order to share good practices and lessons learned. Ideally, enhancing local capacity to implement CSCs should be ensured through the use of existing local structures and the provision of technical support by the implementing partners until the local partners are competent enough to conduct CSC activities including facilitation, negotiation, mediation and community mobilization.

List of Abbreviations

CSC	Community Scorecard
DHO	District Health Office
DHT	District Health Team
FGDs	Focus group discussions
FHS	Future Health Systems

HDREC	Higher Degrees Research and Ethics Committee
HUMCs	Health Unit Management Committees
KIIs	Key Informant interviews
LC	Local council
MakSPH	Makerere University School of Public Health
NGO	Non-Governmental Organization
RMNCAH	Reproductive Maternal Newborn Child and Adolescent Health
SAS	Senior Assistant Secretary
UNCST	Uganda National Council of Science and Technology
VHTs	Village Health Teams
WHO	World Health Organization

Declarations

Ethical consideration

Ethical clearance was obtained from the Makerere University School of Public Health Research and Ethics Committee (MakSPH HDREC) and the Uganda National Council of Science and Technology (UNCST), study number SS 4323. Permission to carry out the research was further sought from the Kibuku District Health Office. The objectives, benefits and risks of the study were explained to the study participants and written informed consent obtained from all the

participants of the FGDs and KIIs. The participants were also informed that their participation in the study was voluntary.

Consent for publication

Consent to publish the findings of the study was obtained at the point of seeking consent to participate. The participants' confidentiality and anonymity while reporting was assured with only investigators and the research team having access to the transcripts.

Availability of data and materials

The data used to undertake this study can be availed on request from the corresponding author.

Competing interests

All the authors had no competing interests

Funding

The project was supported by DFID through the Future Health Systems (FHS) Consortium program under the Award Number HRPC09 Delivering Effective Health Services. The content of this manuscript is solely the responsibility of the authors and does not necessarily represent the official views of the DFID. We also acknowledge the Kibuku district administration, the research assistants and our study participants.

Authors' contributions

All authors contributed towards the conceptualization of the paper. EKK and CA did the data coding, conducted the analysis for the study and led the drafting of the manuscript. All authors reviewed the drafts, provided significant intellectual input and approved the final manuscript.

Acknowledgements

Special thanks to DFID who provided funding for this work through the Future Health Systems consortium. We also appreciate Kibuku Local government and the study participants for taking part in this study.

References

1. Agarwal S, Heltberg R, Diachok M. Scaling-up social accountability in World Bank operations [Internet]. The World Bank; 2009 May [cited 2019 May 23] p. 1–12. Report No.: 51469. Available from: http://documents.worldbank.org/curated/en/423211468164948681/Scaling-up-social-accountability-in-World-Bank-operations

2. World Bank. World Development Report 2004 : Making services work for poor people - Overview [Internet]. The World Bank; 2003 Sep [cited 2019 May 23] p. 1–36. Report No.: 26886. Available from: http://documents.worldbank.org/curated/en/527371468166770790/World-Development-Report-2004-Making-services-work-for-poor-people-Overview

3. Rabbani F, Lalji NS, Abbas F, Jafri SW, Razzak AJ, Nabi N, et al. Understanding the context of balanced scorecard implementation: a hospital-based case study in Pakistan | Implementation Science | Full Text. Implementation Science [Internet]. 2011 [cited 2019 May 21];6(31). Available from: https://implementationscience.biomedcentral.com/articles/10.1186/1748-5908-6-31

4. Blake C, Annorbah-Sarpei NA, Bailey C, Ismaila Y, Deganus S, Bosomprah S, et al. Scorecards and social accountability for improved maternal and newborn health services: A pilot in the Ashanti and Volta regions of Ghana. Int J Gynaecol Obstet. 2016 Dec;135(3):372–9.

5. Peters HD, Noor AA, Lakhwinder PS, Kakar KF, Hansen MP, Burnham G. A balanced scorecard for health services in Afghanistan A balanced scorecard for health services in Afghanistan. Bulletin of the World Health Organization. 2007; 85:146–51.

6. Mutale W, Stringer J, Chintu N, Chilengi R, Mwanamwenge MT, Kasese N, et al. Application of Balanced Scorecard in the Evaluation of a Complex Health System Intervention: 12 Months Post Intervention Findings from the BHOMA Intervention: A Cluster Randomised Trial in Zambia. PLOS ONE. 2014 Apr 21;9(4): e93977.

7. Weir E, d'Entremont N, Stalker S, Kurji K, Robinson V. Applying the balanced scorecard to local public health performance measurement: deliberations and decisions. BMC Public Health. 2009 May 8; 9:127.

8. Edward A, Osei-Bonsu K, Branchini C, Yarghal T shah, Arwal SH, Naeem AJ. Enhancing governance and health system accountability for people centered healthcare: an exploratory study of community scorecards in Afghanistan. BMC Health Services Research. 2015 Jul 31;15(1):299.

9. Ho LS, Labrecque G, Batonon I, Salsi V, Ratnayake R. Effects of a community scorecard on improving the local health system in Eastern Democratic Republic of Congo: qualitative evidence using the most significant change technique. Confl Health [Internet]. 2015 Sep 3 [cited 2019 May 23];9. Available from: https://www.ncbi.nlm.nih.gov/pmc/articles/PMC4557760/

10. Joshi A. Do They Work? Assessing the Impact of Transparency and Accountability Initiatives in Service Delivery. Development Policy Review. 2013;31(s1):s29–48.

11. Fox J. Social Accountability: What Does the Evidence Really Say? GPSA Working Paper No. 1 [Internet]. Washington DC: Global Partnership for Social Accountability; 2014 Sep. Available from: http://gpsaknowledge.org/wp-content/uploads/2014/09/Social-Accountability-What-Does-Evidence-Really-Say-GPSA-Working-Paper 1.pdf

12. Guerzovich F, Moses M. Learning To Open Government; Findings and reflections on how the Open Government Partnership is Playing Out, in Practice, in Five Countries. Washington DC: Global Integrity; 2016.

13. Brockmyer B, Fox J. Assessing the Evidence: The Effectiveness and Impact of Public Governance-Oriented Multi-Stakeholder Initiative [Internet]. London: Transparency and Accountability Initiative; 2015. Available from: https://www.transparency-initiative.org/blog/429/assessing-the-evidence-the-effectiveness-and-impact-of-public-governance-oriented-multi-stakeholder-initiatives/

14. Ekirapa-Kiracho E, Paina L, Kiwanuka NS, Aanyu C, Apolot RR, Bennett S. Community score cards and citizen report cards in Uganda ; What facilitates and constrains implementation? (upcoming). International Journal for Equity in Health. 2019;

15. Simmons R, Fajans P, Ghiron L. Scaling up health service delivery: from pilot innovations to policies and programmes [Internet]. Geneva: Geneva : World Health Organization; 2007 [cited 2018 Dec 19]. Available from: http://apps.who.int/iris/handle/10665/43794

16. Milat AJ, King L, Bauman AE, Redman S. The concept of scalability: increasing the scale and potential adoption of health promotion interventions into policy and practice. Health Promot Int. 2013 Sep 1;28(3):285–98.

17. Fox J. Scaling accountability through vertically integrated civil society policy monitoring and advocacy. Brighton: The Institute of Development Studies; 2016.

18. Fox J. Taking scale into account in transparency and accountability initiatives, Research Report Summary [Internet]. Brighton: IDS; 2016 [cited 2019 Jun 25]. Available from: https://scholar.google.com/scholar?hl=en&as_sdt=0%2C5&q=Taking+scale+into+account+in+transparency+and+accountability+initiatives&btnG=

19. Halloran B. Thinking and working politically in the transparency and accountability field [Internet]. 2014 p. 5. Available from: http://www.transparency-initiative.org/wp-content/uploads/2017/03/thinking-and-working-politically-may-2014.pdf

20. Chandy L, Hosono A, Kharas H, Linn J. Getting to Scale: How to Bring Development Solutions to Millions of Poor People. Brookings Institution Press; 2013. 394 p.

21. Wild L, Harris D. The political economy of community scorecards in Malawi. 111 Westminster Bridge Road London SE1: Overseas Development Institute; 2012.

22. Ekirapa-Kiracho E, Namuhani N, Apolot RR, Aanyu C, Mutebi A, Tetui M, et al. Influence of community scorecards on MNH service delivery and utilization (upcoming). International Journal for Equity in Health. 2020;

23. Uganda Bureau of Statistics. National Population and Housing Census 2014; Area Specific Profiles; Kibuku district [Internet]. Kampala, Uganda: Uganda Bureau of Statistics; 2014. Available from: https://www.ubos.org/onlinefiles/uploads/ubos/2014CensusProfiles/KIBUKU.pdf

24. Gullo S, Galavotti C, Altman L. A review of CARE's Community Score Card experience and evidence. Health Policy Plan. 2016 Dec;31(10):1467–78.

25. Ssebagereka A, Mayora C, Nyachwo BE, Ekirapa-Kiracho E. Estimating the cost of implementing a Facility and Community Score Card to Improve Utilization and Quality of Maternal and Newborn Care Services in a rural district in Uganda (Upcoming). International Journal for Equity in Health. 2020;

26. Yamey G. Scaling Up Global Health Interventions: A Proposed Framework for Success. PLOS Medicine. 2011 Jun 28;8(6):e1001049.

27. Victora CG, Barros FC, Assunção MC, Restrepo-Méndez MC, Matijasevich A, Martorell R. Scaling up maternal nutrition programs to improve birth outcomes: a review of implementation issues. Food Nutr Bull. 2012 Jun;33(2 Suppl):S6-26.

28. Hanson K, Ranson MK, Oliveira-Cruz V, Mills A. Expanding access to priority health interventions: a framework for understanding the constraints to scaling-up. Journal of International Development. 2003;15(1):1–14.

29. Kohl R, Cooley L. Scaling Up–A conceptual and operational framework. Washington, DC: Management Systems International. 2003;1–31.

30. World Health Organization. Nine steps for developing a scaling-up strategy; ExpandNet. Geneva/New York: World Health Organization; 2010 p. 42.

31. Bennett S, Peters DH. Future Health Systems Theory of change for the Institutionalization of Community score cards. 2017.

32. WHO. 20 Questions for Developing a Scaling up Case Study MSI ExpandNet. World Health Organization; 2007 Feb.

33. Keenan KF, Van Teijlingen E, Pitchforth E. The analysis of qualitative research data in family planning and reproductive health care. BMJ Sexual & Reproductive Health. 2005;31(1):40–43.

34. Bennett S, Mahmood SS, Edward A, Tetui M, Ekirapa-Kiracho E. Strengthening scaling up through learning from implementation: comparing experiences from Afghanistan, Bangladesh and Uganda. Health Research Policy and Systems. 2017 Dec 28;15(2):108.

35. World Bank. South Asia Sustainable Development Department, World Bank 2007. Social Accountability Series. Note 1. 2007.

36. Joshi A. Reading the Local Context: A Causal Chain Approach to Social Accountability. [Internet]. IDS Bulletin, 45: 23–35; 2014. Available from: http://onlinelibrary.wiley.com/doi/10.1111/1759-5436.12101/abstract

37. Fox J. Accountability Politics: Power and Voice in Rural Mexico: [Internet]. Oxford University Press; 13 December 2007a. Available from: DOI:10.1093/acprof:oso/9780199208852.001.0001

38. Greenhalgh T, Robert G, Macfarlane F, Bate P, Kyriakidou O. Diffusion of innovations in service organizations: systematic review and recommendations. Milbank Q. 2004;82(4):581–629.

39. Musgrave M, Wong S. Towards a More Nuanced Theory of Elite Capture in Development Projects. The Importance of Context and Theories of Power. Journal of Sustainable Development. 2016 May 30;9(3):p87.

40. Norton W, Mittman B. Scaling-up health promotion/disease prevention programs in community settings: barriers, facilitators, and initial recommendations. Report Submitted to Patrick and Catherine Weldon Donaghue Medical Research Foundation. 2010;

Figures

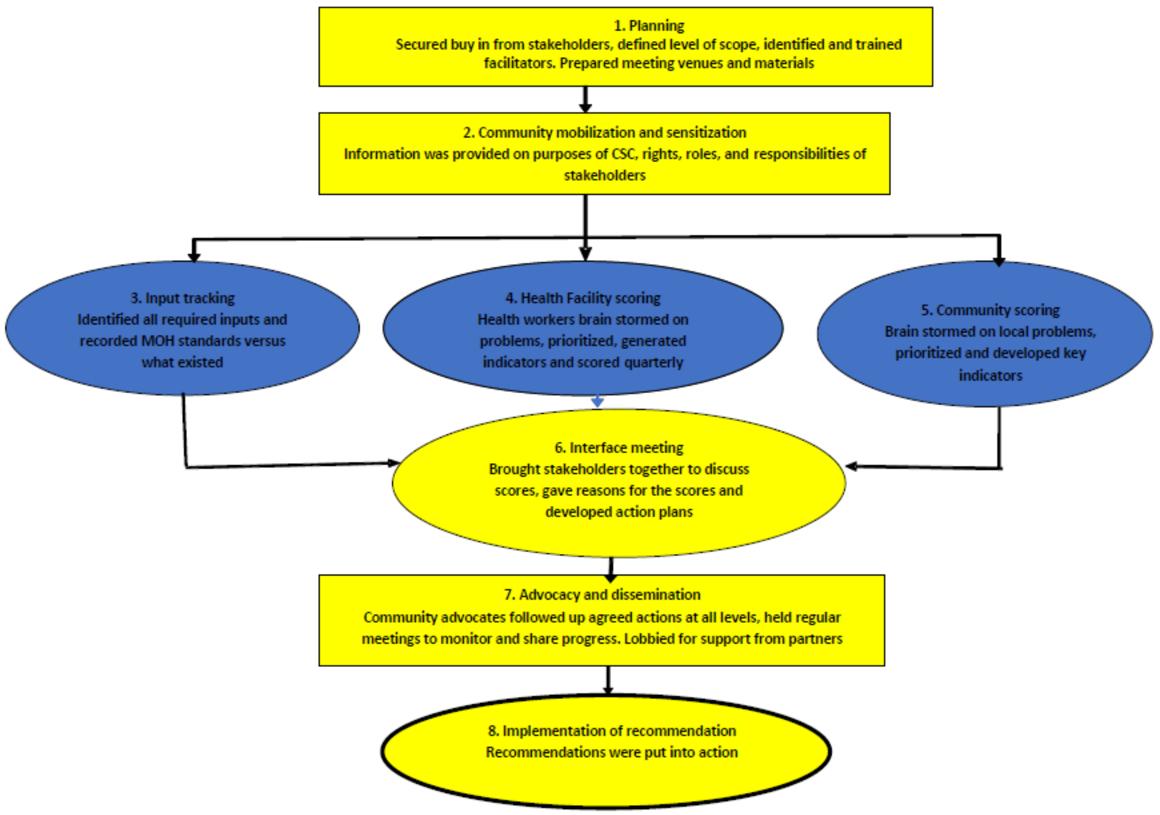

Figure 1: The community score card process (Adapted from the Care CSC)

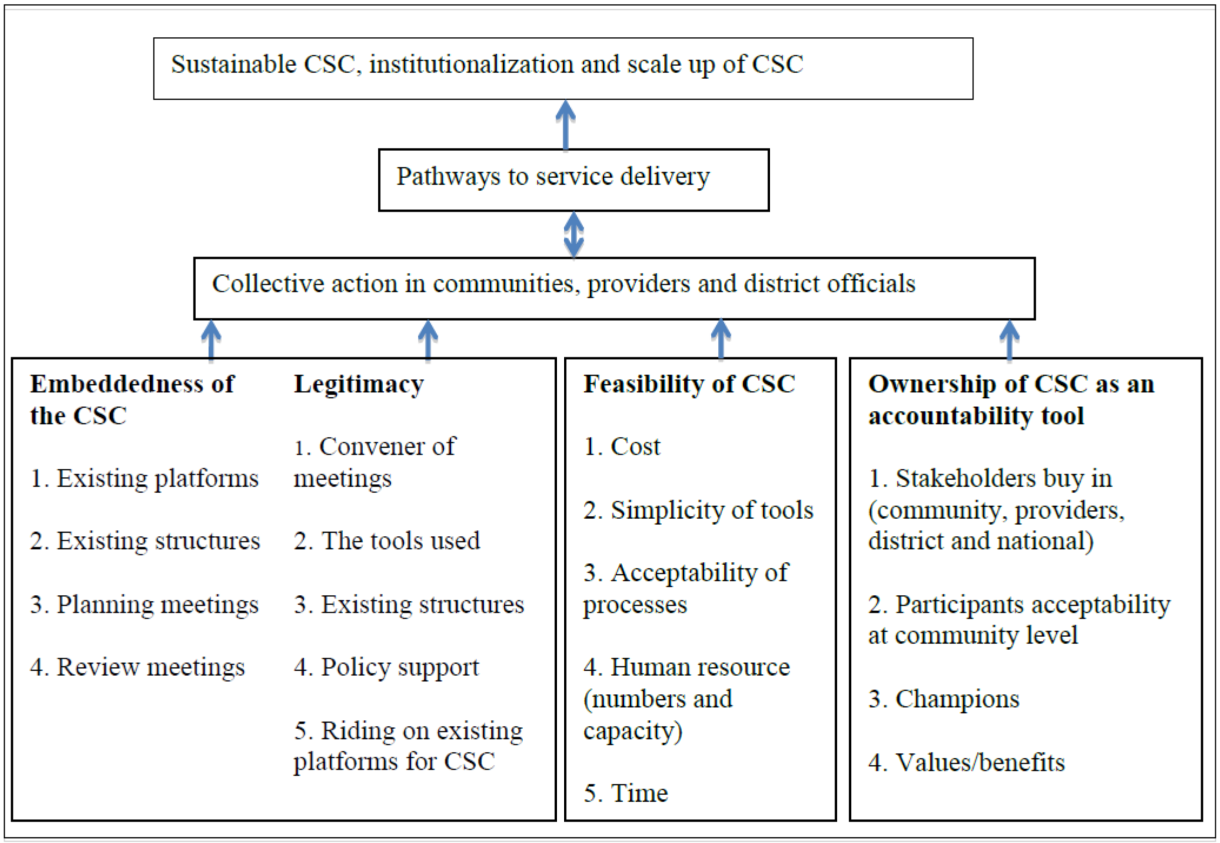

Figure 2: Theory of change
